# Coupled ATPase-adenylate kinase activity in ABC transporters

**DOI:** 10.1038/ncomms13864

**Published:** 2016-12-22

**Authors:** Hundeep Kaur, Andrea Lakatos-Karoly, Ramona Vogel, Anne Nöll, Robert Tampé, Clemens Glaubitz

**Affiliations:** 1 Institute for Biophysical Chemistry and Centre for Biomolecular Magnetic Resonance, Goethe-University Frankfurt, Max-von-Laue-Strasse 9, 60438 Frankfurt am Main, Germany; 2 Institute for Biochemistry, Goethe-University Frankfurt, Max-von-Laue-Strasse 9, 60438 Frankfurt am Main, Germany

## Abstract

ATP-binding cassette (ABC) transporters, a superfamily of integral membrane proteins, catalyse the translocation of substrates across the cellular membrane by ATP hydrolysis. Here we demonstrate by nucleotide turnover and binding studies based on ^31^P solid-state NMR spectroscopy that the ABC exporter and lipid A flippase MsbA can couple ATP hydrolysis to an adenylate kinase activity, where ADP is converted into AMP and ATP. Single-point mutations reveal that both ATPase and adenylate kinase mechanisms are associated with the same conserved motifs of the nucleotide-binding domain. Based on these results, we propose a model for the coupled ATPase-adenylate kinase mechanism, involving the canonical and an additional nucleotide-binding site. We extend these findings to other prokaryotic ABC exporters, namely LmrA and TmrAB, suggesting that the coupled activities are a general feature of ABC exporters.

## Abstract (web summary)

ATP-binding cassette (ABC) transporters hydrolyse ATP to transport molecules across the cell membrane. Here Vogel *et al*. show that the ABC exporter MsBA can couple ATP hydrolyse to an adenylate kinase activity that seems to be predominant at low ATP levels and a general feature of ABC exporters.

ATP-binding cassette (ABC) transporters contribute to important cellular processes by catalysing transport reactions across the membrane involving, for example, protein secretion, nutrient uptake, peptide and drug export as well as lipid translocation by ATP hydrolysis^1^,^2^. They are of utmost medical importance. For example, drug exporters are known to contribute significantly to drug resistance in cancer cells (for example, P-glycoprotein) or to antibiotic resistance in bacteria^3^. In addition, mutations in lipid transporters have been directly linked to a number of human diseases^4^.

The understanding of ABC exporters has been greatly enhanced by the increasing number of structures such as Sav1866 (ref. 5), TM287/288 (ref. 6), TmrAB^7^, MsbA^8^, P-glycoprotein^9^, PglK^10^ and ABCB10 (ref. 11). They share a canonical architecture with two cytosolic nucleotide-binding domains (NBDs) and two transmembrane domains (TMDs). In full transporters, four domains are formed by a single polypeptide chain and in half-transporters, one TMD and one NBD are fused together and a functional protein is formed through dimerization. The TMDs are responsible for substrate translocation. They show little sequence conservation and, in case of exporters, usually consist of six transmembrane helices. NBDs in contrast, which hydrolyse ATP in order to drive substrate translocation, dimerize in a sandwich-like manner and have many conserved sequence motifs. These motifs (A-loop, Walker A, Q-loop, Signature motif, Walker B, D- and H-loop) play an important role in the catalytic activity.

Despite intense structural and biochemical studies on isolated NBDs and known structures of full-length transporters (for reviews see refs 12, 13, 14), many questions remain open. These concern the actual ‘process of ATP hydrolysis' and the cross-talk between NBDs and TMDs during the transport cycle including origin and implication of basal and stimulated ATPase activity in ABC transporters. Current knowledge cumulates in two commonly discussed models. One model suggests switching between a closed NBD dimer with two ATP molecules ‘sandwiched' at the dimer interface and an open, dissociated dimer as a result of hydrolysis of both ATPs (‘processive clamp, also named switch model')^15^,^16^,^17^,^18^. Alternatively, the ‘constant contact model' does not involve NBD dimer dissociation but proposes alternating opening of both ATP-binding sites at the point of sequential ATP hydrolysis^19^,^20^,^21^.

In the present study on the ABC exporter MsbA, we add an additional layer of complexity by providing evidence that MsbA not only depends on its primary reaction ATP hydrolysis but is also able to catalyse an adenylate kinase (AK) reaction that appears most pronounced once ATP levels are low. Both processes can couple in a cyclic way. The possibility of an additional adenylate kinase activity has been described for soluble ABC-type proteins^22^,^23^ as well as for the ABCC7 chloride channel cystic fibrosis transmembrane conductance regulator (CFTR)^24^,^25^ but not yet for ‘conventional' ABC transporters.

MsbA is a homodimeric ABC exporter (130 kDa) from *Escherichia coli* that functions as lipid A flippase^26^,^27^. Lipid A is a component of lipopolysaccharide, an integral moiety in the outer membrane of Gram-negative bacteria^28^. Furthermore, it has been shown that MsbA can translocate a range of hydrophobic substrates such as those transported by multidrug transporters P-glycoprotein and LmrA and has been therefore classified as multidrug exporter^29^. The overlapping substrate specificities between these proteins indicate structural similarity^30^. MsbA from *E. coli*, *Salmonella typhimurium* and *Vibrio cholera* has been crystallized in apo, AMP-PNP bound and ADP-Vi trapped states, respectively^8^. These crystal structures provide an insight into the probable conformational changes involved during ATP hydrolysis and, potentially, substrate translocation. Based on a significant amount of fundamental structural and functional information^21^,^31^,^32^,^33^,^34^,^35^,^36^, MsbA can be considered as model to explore the mechanism of substrate translocation by ABC exporters.

Here we demonstrate that a coupled ATPase-AK reaction can play a role for the functional mechanism of MsbA especially under ATP depletion conditions. Evidence is provided by solid-state NMR based on magic-angle sample spinning (MAS) that allows the structural and mechanistic analysis of full-length membrane proteins directly within proteoliposomes near physiological conditions^37^. In our previous work, we demonstrated that MsbA is well suited for in-depth MAS-NMR analysis based on ^13^C/^15^N spectra as it shows long-term stability and can be isotope labelled and reconstituted into various lipid bilayers^38^. Here, for probing its catalytic activity within the lipid bilayer, we utilize real-time ^31^P-MAS NMR to monitor substrate turnover by recording progress curves for all compounds involved. This approach has been shown to offer unique insight into membrane protein catalysed reactions involving, for example, ATP hydrolysis and phosphoryl group transfers^39^. Furthermore, ^1^H–^31^P cross-polarization experiments are used for identifying transporter-bound nucleotide species. The ^31^P isotope is the ideal probe for fast, label-free and noninvasive observation of reactions because of its large chemical shift dispersion, its high sensitivity and 100% natural abundance as NMR-active nuclei. As demonstrated for diaclygylcerol kinase, coupled reactions at and within the lipid bilayer can be monitored directly and simultaneously^39^. Based on single-point mutations introduced in the Walker A, Signature, D-loop, Q-loop and Walker-B motifs of the MsbA NBDs, a model is proposed in which the canonical nucleotide-binding site, needed for ATP hydrolysis, together with a postulated second site forms the AK reaction centre. Our findings are supported by substrate transport measurements and are extended to other ABC exporters such as LmrA and TmrAB and discussed as a potentially general feature for this ABC exporter subfamily.

## Results

### Nucleotide turnover experiments by real-time ^31^P-MAS NMR

**Figure 1:**
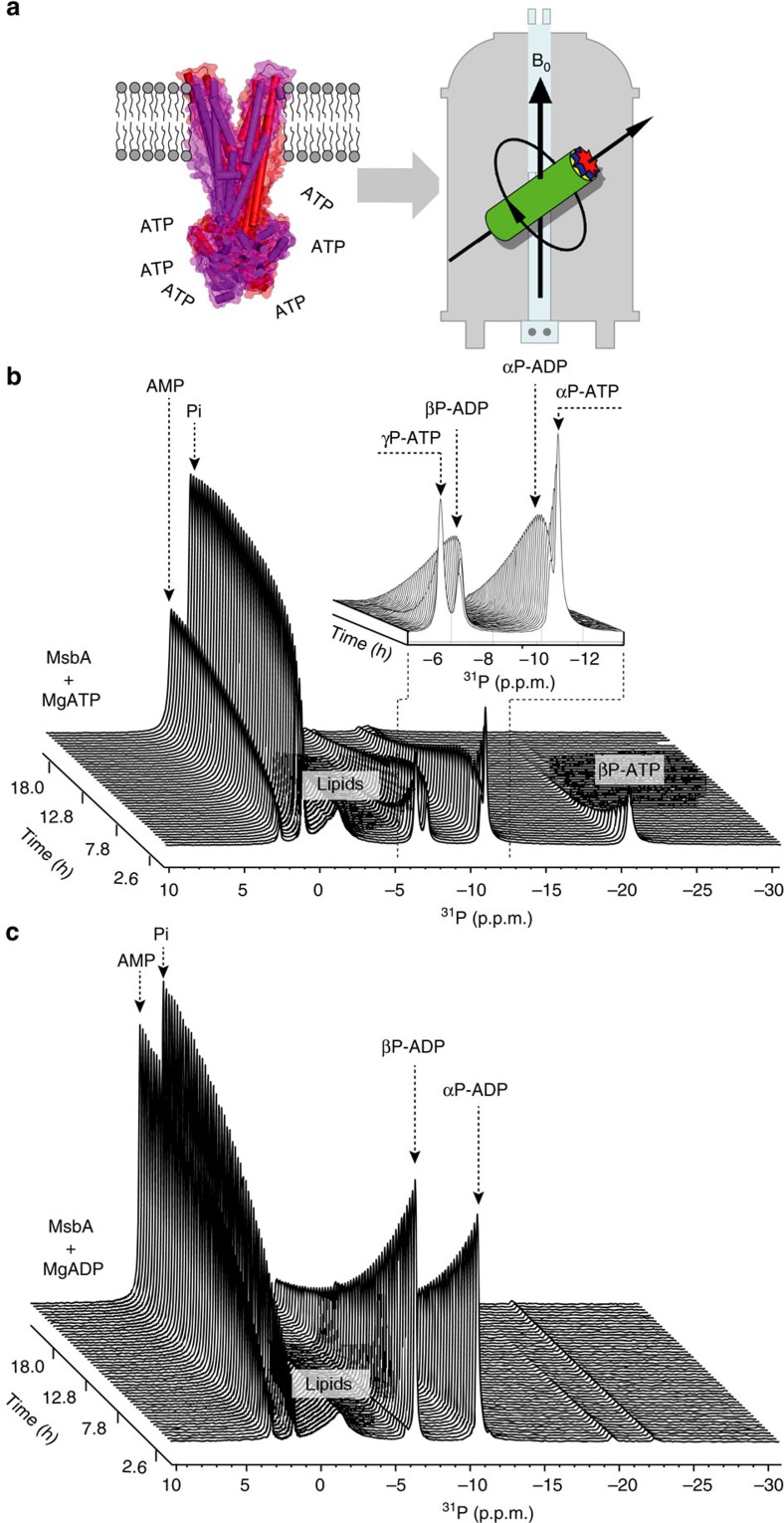
Probing the catalytic activity of MsbA by real-time ^31^P-MAS NMR. (**a**) Schematic experimental setup. MsbA is transferred quickly within the MAS rotor to the NMR magnet after addition of MgATP. The catalytic activity is followed by direct polarization ^31^P NMR. (**b**) Time-resolved spectra show the reduction of the ATP peak intensities (αP, βP and γP at −10.89, −20.48 and −6.22 p.p.m., respectively) and the increase of Pi (1.05 p.p.m.) and AMP (2.52 p.p.m.). The ADP resonance intensities (αP and βP at −10.68 and −7.4 p.p.m.) follow a biphasic time course with initial increase and subsequent decay (inset). (**c**) Addition of MgADP to MsbA proteoliposomes results in a reduction of the ADP resonances. The ratio of integral peak intensities of Pi/AMP approaches 2:1 in **b** and 1:1 in **c**.

The basic experimental setup is shown in Fig. 1a. MgATP was added to MsbA proteoliposomes, the sample was quickly transferred into the MAS probe and ^31^P detection started within a dead time of 5 min. An initial molar stoichiometry of MgATP/MsbA of 16:1 was used that was completely consumed during the experiment so that progress curves of all other nucleotide species involved could be recorded. Specific conditions were selected under which the reactions run slower so that a better signal-to-noise ratio for each data point as well as a better sampling of the NMR progress curves could be achieved and under which best spectral resolution is obtained (non-saturation levels of ATP, lower temperature, ATP/Mg^2+^ 3:1). All experimental details and related control experiments are described in the Methods section. Because of the nature of the experiment, *in vitro* conditions deviate of course from the levels found *in vivo* where other additional nucleotide species are also simultaneously present. The *in vivo* concentrations are dependent on various factors such as stress conditions, stage of cell growth or division and so on.

A typical pseudo-two-dimensional time-resolved data set is shown in Fig. 1b. As expected, during the course of the experiment, the signal intensities of the αP, βP and γP resonances of ATP reduce, whereas αP and βP of ADP and inorganic phosphate appear because of ongoing ATP hydrolysis. Under these conditions ATP gets consumed completely during the course of the reaction. A distinct biphasic behaviour for both ADP signals is observed as their initially increasing intensity reduces again with time (inset, Fig. 1b). In addition, a new resonance assigned to AMP appears (Fig. 1b). The build-up of both products (AMP and Pi) occurs monotonically with highly reproducible final stoichiometry of 2:1 for Pi/AMP. These observations suggest that MsbA is consuming not only ATP but also ADP. Indeed, addition of only ADP to MsbA results in its complete conversion into AMP and Pi at a final stoichiometry of 1:1 (Fig. 1c).

These data indicate that MsbA is not only able to catalyse ATP hydrolysis but there is a possibility of a second reaction involving consumption of ADP either via


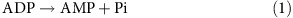


or


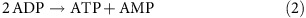


For both cases, because of the simultaneously occurring ATP hydrolysis, a similar kinetics of the ADP, AMP and Pi resonances in time-resolved ^31^P-spectra and the same 2:1 stoichiometry of Pi/AMP is expected. Therefore, the only way to differentiate between both reactions would be to detect whether formation of ATP takes place or not. The data in Fig. 1c do not explicitly show ATP formation from ADP. They do not offer unambiguous evidence for scheme (1) either as any newly formed ATP might get hydrolysed right away without being released from MsbA, making its detection difficult.

**Figure 2:**
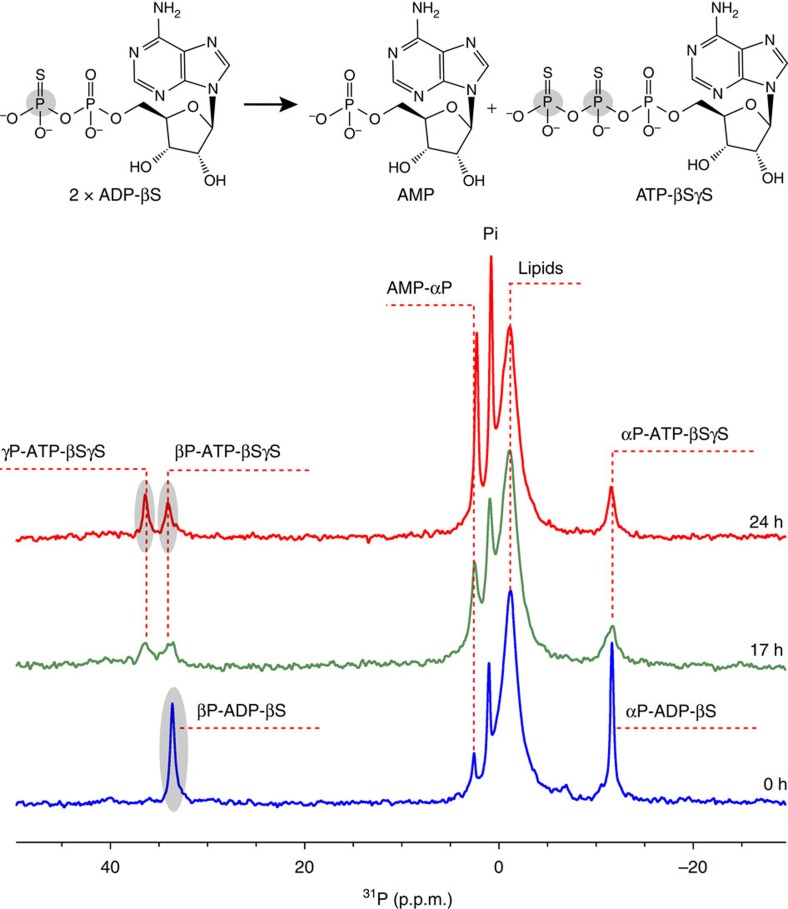
Evidence for ATP synthesis by MsbA. ADP-βS is converted into AMP and ATP-βSγS, a non-hydrolysable ATP analogue. The αP and βP chemical shifts of ADP-βS are observed at −11.5 and 33.7 p.p.m., respectively. With time, the ADP-βS signals decay and the formation of ATP-βSγS is observed with signals at −11.5, 34.22 and 36.25 p.p.m. corresponding to αP, βP and γP, respectively.

Therefore, an experiment was conducted in which immediate hydrolysis of potentially generated ATP is prevented by offering ADP-βS as the sole nucleotide to MsbA. Reaction scheme (1) would result in AMP and S-Pi, whereas scheme (2) would produce non-hydrolysable ATP-βSγS and AMP (Fig. 2). An additional advantage is that thiophosphate substitution results in a significant ^31^P chemical shift change allowing better discrimination between reaction products containing S or O. The spectra in Fig. 2 clearly show reduction of the ^31^P resonances of ADP-βS. Formation of inorganic thiophosphate (S-Pi), which is expected to appear at 44.5 p.p.m. (ref. 39), cannot be observed. Instead, two new resonances corresponding to the βP and γP positions of ATP-βSγS appear proving that the protein follows reaction scheme (2), a reverse adenylate kinase reaction.

**Figure 3:**
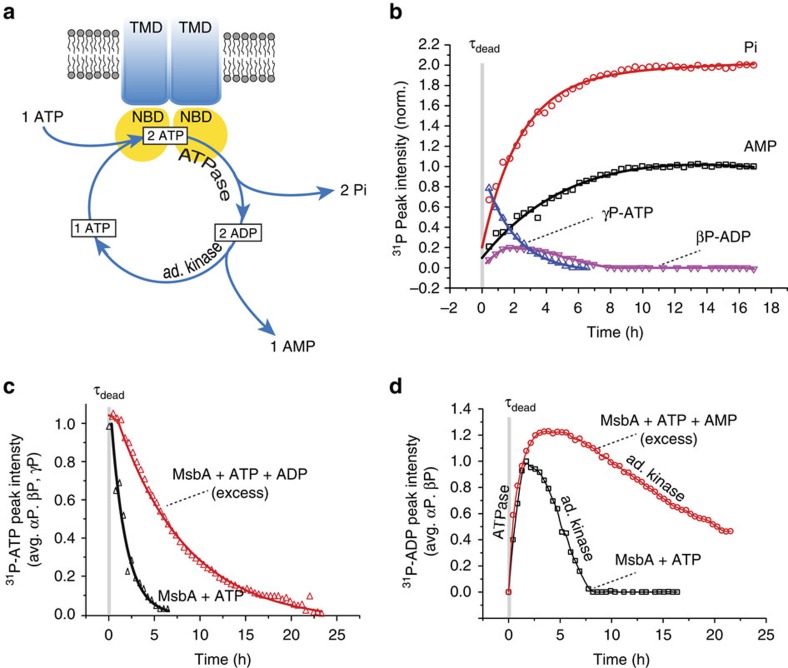
Proposed model for a coupled ATPase-AK reaction. (**a**) Hydrolysis turns ATP into ADP and Pi, followed by conversion of both ADP into AMP and ATP. (**b**) This coupled catalytic activity results in ^31^P progress curves for each nucleotide species obtained by deconvoluting the data set in Fig. 1b. The observed integral peak intensities correspond to a stoichiometry of ATP at the beginning of the reaction AMP and Pi at the end of 1:1:2. (**c**) The progress curve for the consumption of ATP decays slower if ADP is added in excess in addition to ATP. (**d**) The ATPase activity of MsbA results in an initial positive slope of the ADP progress curve, whereas its kinase activity causes a decrease. The addition of excess AMP in addition to ATP has a small effect on the initial increase but significantly slows down ADP consumption. Full data sets for (**c**,**d**) are provided in Supplementary Fig. 11.

Our data show that MsbA not only hydrolyses ATP but can also catalyse a reverse adenylate kinase reaction. Both reactions are observed simultaneously in our experimental setup and appear to consume each other's products. The AK reaction, as monitored by the AMP generation, appears most pronounced under conditions of low amounts of ATP, when high levels of ADP have been produced. Therefore, we propose a cyclic and coupled ATPase-AK mechanism for MsbA that is depicted in Fig. 3a: MsbA hydrolyses 2ATP into 2ADP and 2Pi molecules. Subsequently, 2ADP are converted into AMP and ATP. The ‘regenerated' ATP enters again the hydrolysis cycle together with one additional ATP taken up from solution. This scheme explains the progress curves for ATP, ADP, Pi and AMP peak intensities shown in Fig. 3b that have been obtained by deconvoluting the data set from Fig. 1b: the observed stoichiometric ratios obtained from the observed integral peak intensities of ATP at the beginning of the reaction to AMP and Pi at the end is 1:1:2 as expected from the cycle in Fig. 3a. ADP displays a biphasic progress curve with a positive slope governed mostly by the ATPase and a negative slope dominated by the AK mechanism of MsbA. The build-up of AMP starts with the generation of Pi, proving that both reactions indeed run simultaneously. Progress curves obtained by deconvolution of the data set in Fig. 1c (addition of MgADP) result in a stoichiometric ratio of ADP at the beginning of the reaction to AMP and Pi at the end of 1:1:1, as expected from the coupled ATPase-AK cycle (Supplementary Fig. 1a,b).

Proposing such a reaction scheme raises questions about numbers and location of nucleotide-binding sites, because two ADP molecules must bind at the same time in close proximity with respect to each other for the kinase reaction to take place. We have therefore explored whether addition of excess ADP or AMP affects either the ATPase or the kinase reaction or both.

In the presence of excess ADP, the consumption of ATP is slowed down and follows a more complex multi-exponential progress curve (Fig. 3c). There are two possible explanations: (1) the reverse adenylate kinase reaction (2ADP→ATP+AMP) converts the additional ADP into a large ATP reservoir that takes longer to get hydrolysed or (2) excess ADP competes with ATP for binding to the canonical binding site that would reduce the ATPase activity. Of course both processes could contribute simultaneously. The progress curve for ADP in the presence of excess AMP (Fig. 3d) shows significantly slower consumption of ADP reflective of the kinase reaction. In addition, more ADP is produced in the initial phase at an apparent rate similar to that in the absence of AMP. This observation could be explained by (1) a forward adenylate kinase reaction (ATP+AMP→2ADP) and/or (2) by competition of excess AMP with ADP for binding. A reverse adenylate kinase reaction has been observed for the *Pyrococcus furiosus* structural maintenance of chromosome protein (*pf*SMC)^23^, whereas both reverse and forward reactions have been reported for CFTR^24^. Both effects of excess ADP and AMP indicate the existence of an additional binding site in addition to the canonical site.

### Detecting nucleotide bound to MsbA by ^31^P-MAS NMR

**Figure 4:**
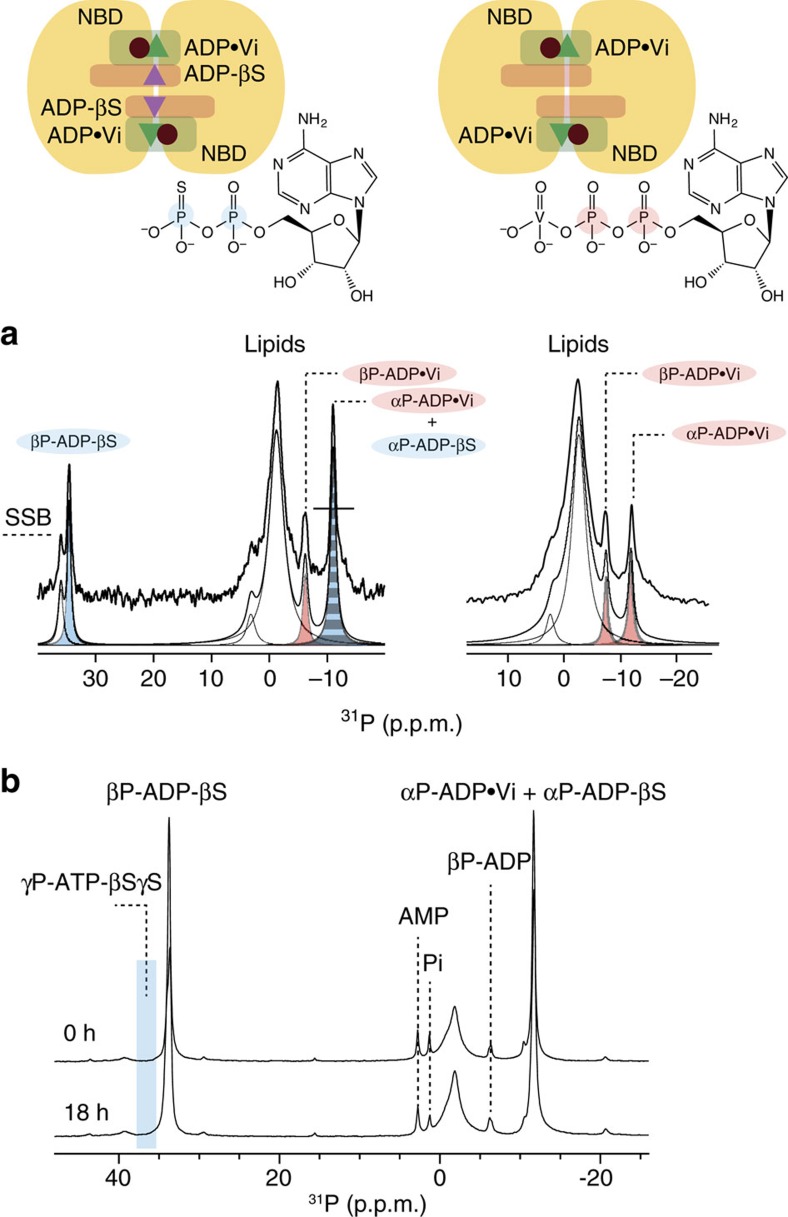
ADP.Vi-trapped MsbA with bound ADP-βS. (**a**) Cross-polarized (CP) ^31^P-MAS NMR allows to observe bound ADP.Vi at −6.2 and −10.9 p.p.m. (right). Upon addition of excess of ADP-βS, an additional resonance at 34.5 p.p.m. corresponding to βP-ADP-βS is observed. Furthermore, the peak at −11.0 p.p.m. doubles because of the overlap of αP-ADP-βS and aP-ADP.Vi. Peak deconvolution reveals the same integral peak intensities for ADP-βS and ADP.Vi, indicative of a 1:1 stoichiometry. (**b**) Directly polarized ^31^P-MAS NMR spectra of MsbA in complex with ADP-βS and ADP.Vi shows that no γP-ATP-βSγS is produced as shown in Fig. 2. Spectra were recorded under the same conditions as described in the Methods. The nominal temperature in **a** was set to 260 K and in **b** to 270 K. SSB refers to spinning sideband.

The experiments described above are based on direct ^31^P excitation, by which turnover of all nucleotide species is observed. In order to filter just the signal of nucleotides bound to the NBDs of MsbA, we utilized dipolar-based cross-polarization (CP) magnetization transfer from the homogeneous ^1^H network to ^31^P (ref. 40). In this way, one can discriminate between MsbA-bound from free nucleotides. The later will not cross-polarize and do not appear in the spectra. However, one requirement for detecting bound nucleotides is that the lifetime of the bound state should be long enough for the CP transfer to take place. We therefore trapped MsbA in a catalytic transition state using ADP-Vi. In this state, based on known structural data^8^, we assume both canonical binding sites to be occupied (Fig. 4a, right). Two CP resonances arising from αP- and βP-ADP-Vi can be observed (Fig. 4b, right). A similar spectrum is obtained when trapping a pre-hydrolysis state using ADP-BeFx (Supplementary Fig. 2b). Control experiments confirm that the observed signals are specific for MsbA (Supplementary Fig. 2f–h). Upon addition of excess ADP-βS, an additional signal for βP-ADP-βS is observed (Fig. 4b, left). The βP-ADP·Vi peak remains unchanged, but αP-ADP·Vi doubles because of overlap with αP-ADP-βS. Peak deconvolution and integration confirms that ADP·Vi remains unchanged upon ADP-βS binding and both nucleotides bind with a 1:1 stoichiometry and ADP-βS is not competing with ADP·Vi for binding. If ADP·Vi occupies both canonical binding sites in this homodimer, ADP-βS must therefore bind to two additional sites as indicated in the left of Fig. 4a.

Further support is provided by time-resolved direct-polarized ^31^P-MAS NMR. Offering ADP-βS to apo-MsbA results in ATP-βSγS formation (Fig. 2). However, the addition of ADP-βS to MsbA in a Vi-trapped transition state did not yield an observable reaction (Fig. 4c) although ADP-βS binds to MsbA. This means that the binding sites occupied by ADP-βS in this Vi-trapped sample do not allow both nucleotides to come close enough for the kinase reaction to occur. A possible explanation would be that in apo-MsbA, ADP-βS could occupy both the canonical and an additional binding site within each NBD leading to ATP-βSγS formation as shown in Fig. 2, whereas in Vi-trapped MsbA, ADP-βS could only bind to the additional site. The absence of an observable reaction in Fig. 4c also serves as a control for complete trapping: if just a certain fraction of MsbA would have been trapped and ADP-βS would bind to the remaining MsbA population, a reaction as in Fig. 2 should have been observed.

### Probing catalytic centre of MsbA by single-point mutations

**Figure 5:**
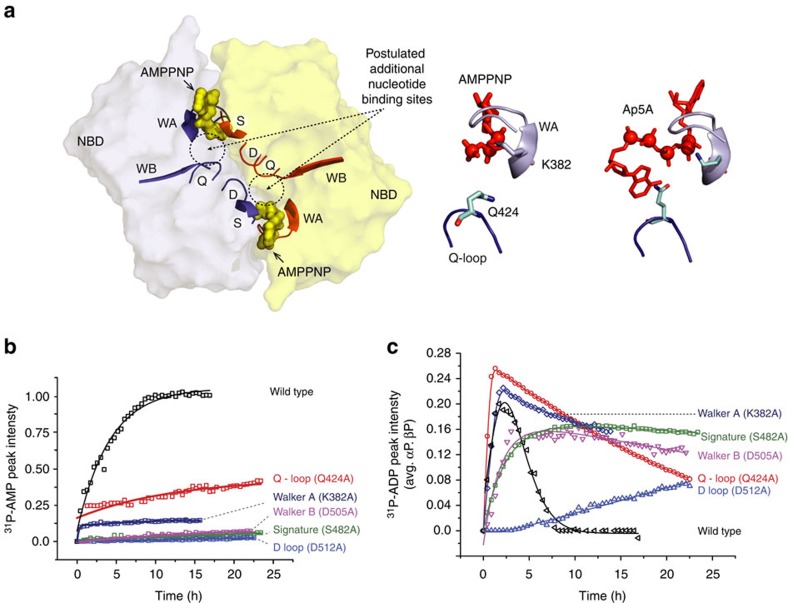
Mutational analysis of the coupled ATPase-AK activity of MsbA. (**a**) Visualization of the conserved NBD sequence motifs in the MsbA/AMP.PNP crystal structure (pdb: 3B60). The canonical binding sites and a second postulated binding sites are highlighted. Key residue K382 in Walker A is in close proximity to the nucleotide phosphate groups (top middle). In pfSMC^NBD^, the Q-loop glutamine was found coordinating binding of Ap5A indicative of a second binding site (top right, pdb: 3KTA). All single-point mutations are summarized in Table 1. (**b**) Progress curves of the ^31^P-AMP peak intensities demonstrate much reduced generation of AMP. The initial data points of Q424A are missing due to the experimental dead time. (**c**) Progress curves of the ^31^P-ADP peak intensities are sensitive to both the ATPase (positive slope) and kinase (negative slope) reactions. The mutation Q424A causes accelerated ADP generation (ATPase) but slower consumption (kinase) with respect to wild-type MsbA. The ADP consumption is even slower in case of K382A. The S482A and D505A mutations slow down both ATPase and kinase activity. The D512A mutation causes the slowest ADP generation, whereas no consumption of ADP could be seen on the time scale of the experiment. Experiments were done in triplicate with protein samples from different expressions each time.

In order to identify an additional binding site, single-point mutations were introduced into conserved NBD sequence motifs, depicted in Fig. 5a (left) for MsbA in complex with AMP-PNP^8^. Two nucleotides bind at the NBD dimer interface to binding sites formed by the Walker A motif of one and the signature motif of the opposing NBD. Other conserved motifs, namely Walker B, Q-loop and D-loop, are in close proximity to each other and also near the dimer interface but do not have any nucleotide between them. Walker A is also found in other nucleotide-binding proteins such as adenylate kinase, uridylate kinase or ras A^41^,^42^ and seems to play an important role for their functional mechanism. Walker B is also found in Adk6 that is able to catalyse both ATPase and adenylate kinase activities^43^.

The lysine residue of the Walker A region (MsbA K382) has been shown to have a strong effect on the activity of ABC transporters and kinases^41^,^44^. In the MsbA crystal structure, its side chain points towards the γ and β phosphate of the bound nucleotide (see inset Fig. 5a, middle). In addition, the serine in the Signature motif (MsbA S482) is found in close proximity to the bound nucleotide. Mutations of both residues have been described to cause a significantly reduced activity in ABC transporters^45^,^46^. The Walker B motif supports the coordination of the magnesium ion via the conserved aspartate (MsbA D505)^45^. The Q-loop contains a conserved glutamine residue (MsbA Q424) that switches in and out of the active site during the ATP hydrolysis cycle apart from being one of the sites of interaction with TMDs^4^. In addition, it was found to be involved in the coordination of Ap5A binding in pfSMC^NBD^ indicative of an AMP-binding site (see inset Fig. 5a, right)^23^. The D-loop aspartate (MsbA D512) is known to allosterically control ATP hydrolysis by completing the coordination of water through Mg^2+^ with Walker B and Q-loop and plays a role for positioning Walker A^45^,^47^,^48^,^49^,^50^. We have therefore introduced the single-point mutations K382A (Walker A), Q424A (Q-loop), S482A (signature), D505A (Walker B) and D512A (D-loop) in order to probe their effect onto the coupled ATPase-AK activity of MsbA (see Table 1). A convenient readout for the kinase reaction is provided by the progress curves of the ^31^P-AMP signal intensities, whereas the temporal evolution of the ^31^P-ADP resonance reports on both ATPase and AK reactions.

Our data reveal that the mutations Q424A and K382A slow down the build-up of AMP, whereas in S482A, D505A and D512A almost no AMP generation could be detected (Fig. 5b). A more differentiated view is provided by the ^31^P-ADP progress curves (Fig. 5c). The observed initial signal increase is governed by ATP hydrolysis that is followed by signal reduction dominated by the kinase reaction, but both processes contribute to the positive and negative slopes of the ADP progress curves. In case of Q424, the initial increase is faster and the subsequent signal decay becomes slower compared with wild-type MsbA. This means that this mutation probably reduces the kinase reaction causing a faster apparent rate for ADP generation and/or also causes a faster ATPase activity. The ADP consumption appears even slower for K382A in agreement with the analysis of the AMP progress curves, but in contrast to Q424A, ADP generation is not accelerated. For all other mutations, ADP generation, consumption and AMP production slow down significantly, with the strongest effect caused by D512A followed by S482A and D505A. All observations were verified by also analyzing ^31^P-progress curves for all mutants upon addition of Mg.ADP (Supplementary Fig. 1c,d).

These mutational data therefore provide evidence that the conserved NBD sequence motifs not only stabilize the canonical binding site and control ATP hydrolysis, but must also be involved in the kinase reaction. It seems that especially the Q-loop glutamine Q424 and Walker A lysine K382 play a prominent role that is also evident from crystal structures of MsbA with AMP.PNP^8^ and *pf*SMC with Ap5A (ref. 51) (Fig. 5a middle/right).

### The coupled ATPase-AK mechanism and substrate transport

In order to probe the functional role of the observed ATPase-AK mechanism, substrate transport has been analysed. As MsbA transports not only lipid A but also the substrate Hoechst-33342 (ref. 52), we were able to utilize a well-established transport assay based on fluorescence spectroscopy^52^,^53^,^54^,^55^.

**Figure 6:**
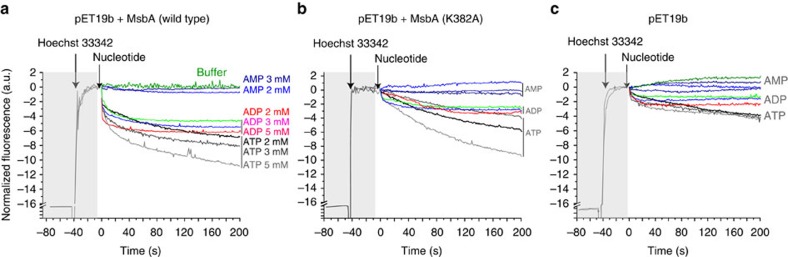
Transport of Hoechst-33342 by MsbA into *E. coli* ISOVs probed by fluorescence spectroscopy. (**a**) ISOVs containing MsbA show a concentration-dependent substrate transport for ATP. Upon addition of AMP, no transport is detected. However, increasing amounts of ADP also lead to transport. (**b**) ISOVs containing the MsbA K382A mutant show much slower but still ATP concentration-dependent transport. The effects in the presence of ADP are much less pronounced and within the range observed for ISOVs prepared from *E. coli* induced with an empty vector (**c**). The latter shows almost no ATP concentration-dependent transport. The small, observed effects could be because of the native *E. coli* ABC transporters and other proteins in the membrane of *E. coli*. All experiments were repeated in triplicate.

The inside-out vesicles (ISOVs) containing MsbA were prepared from *E. coli* cells. Upon addition of increasing concentrations of ATP (2–5 mM), a decrease of the fluorescence quantum yield of Hoechst-33342 with time due to substrate transport into the vesicles was observed (Fig. 6a). Upon addition of AMP, no transport took place. However, a concentration-dependent transport could also be detected for ADP. The experiment was repeated for the K382A mutant of MsbA (Fig. 6b). Overall transport is reduced and the effect of ADP is much less pronounced compared with Fig. 6a.

As a control, the experiment was also performed on ISOVs prepared from *E. coli* cultures in which an empty vector was induced. Here, much less nucleotide concentration-dependent fluorescence changes were detected (Fig. 6c). The remaining activity is probably because of natively occurring transporters. These data demonstrate that ADP can also drive substrate transport because of the coupled ATPase-AK mechanism. This phenomenon is reduced in K382A mutant where the kinase reaction is compromised, in accordance to the data shown in Fig. 5c.

### Observing coupled ATPase-AK activity in other ABC exporters

The fact that ABC transporters share similar NBDs with highly conserved sequence motifs raises the question of whether the coupled ATPase-AK activity is a general feature at least for prokaryotic ABC exporters. We have therefore applied the same experimental ^31^P-MAS NMR approach described here for MsbA to two more ABC exporters, namely LmrA from *Lactococcus lactis* and TmrAB from *Thermus thermophilus*. LmrA is a homodimer sharing the same topology as MsbA and overlapping substrate specificity^29^. TmrAB is a functionally asymmetric heterodimer with one degenerate ATP hydrolysis site^7^,^55^,^56^.

**Figure 7:**
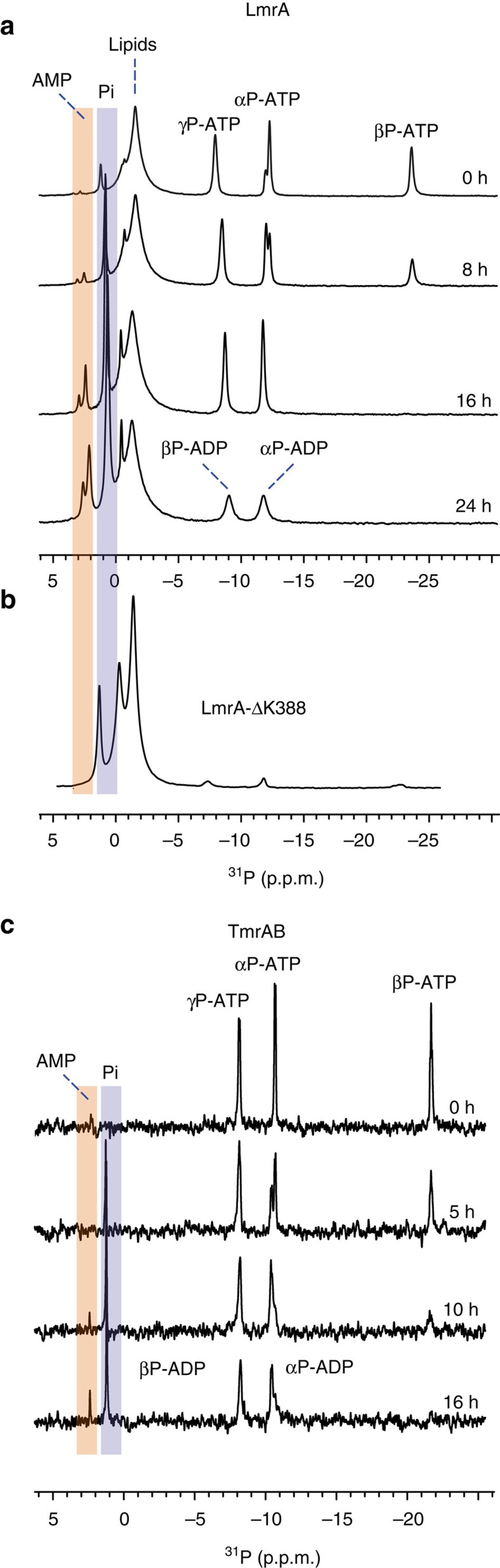
Time-resolved ^31^P-NMR spectra of LmrA and TmrAB in the presence of ATP reveal AMP formation as observed for MsbA. (**a**) ^31^P-MAS NMR spectra of LmrA proteoliposomes (DMPC/DMPA) show that ATP and ADP are consumed over time to yield Pi and AMP, the latter being the kinase activity marker. (**b**) AMP formation is not observed in the Walker A Lysine deletion mutant LmrA-ΔK388. This spectrum, recorded at the end of the reaction, was reported by our lab before and is shown here only for comparison^57^. The slightly different Pi chemical shifts in **a**,**b** are because of the choice of different buffers. (**c**) ^31^P-solution state NMR spectra of TmrAB in detergent micelles (DDM). At 341 K, formation of Pi and AMP is observed over time. The apparently reduced formation of AMP could be contributed to the heterodimeric nature of TmrAB and its lack of one canonical site for ATP hydrolysis.

In case of LmrA, time-resolved ^31^P-MAS NMR spectra indeed demonstrate the conversion of ATP and ADP into AMP and Pi, as shown for MsbA (Fig. 7a). Remarkably, an earlier NMR study from our lab on LmrAΔK388 (ref. 57) a Walker A Lysine deletion mutant, did not show any AMP formation (Fig. 7b). This agrees with our observation of a reduced kinase activity in the Walker A K382A mutant of MsbA and emphasizes the role of this residue in the coupled activity of these transporters. As for MsbA and LmrA, a ^31^P-AMP resonance is also detected in ^31^P-NMR spectra of TmrAB recorded at 68 °C upon addition of ATP (Fig. 7c). The relative Pi and AMP peak intensities in both cases appear smaller compared with MsbA because different amounts of protein were used and the end point of the reaction has not been reached. The reaction seems to run slower in case of TmrAB as it contains one degenerate canonical site. These observations indicate that prokaryotic ABC exporters might be able to catalyse an AK reaction that can be coupled to ATP hydrolysis, irrespective of whether one or two canonical ATP hydrolysis sites exist.

## Discussion

The data presented here demonstrate that the ABC exporter MsbA embedded within lipid bilayers is able to catalyse an adenylate kinase reaction in addition to its primary reaction ATP hydrolysis. Both reactions occur simultaneously and appear strongly coupled under our experimental conditions. The AK reaction becomes most pronounced once ATP got consumed and larger amounts of ADP have been produced. In our experiment, substrate turnover is monitored by detecting ^31^P-NMR progress curves for all nucleotide species involved that results in different levels of ATP, ADP, AMP and Pi at each time point. Evidence for a cyclic coupling of both ATPase and AK reactions comes from our ADP turnover experiments. If the AK activity would not be coupled to the ATPase, MsbA would simply produce ATP if ADP would be offered as substrate. This is however only the case if the ATPase activity is blocked as demonstrated in Fig. 2 (see also Supplementary Fig. 1).

We have further demonstrated that both reactions are associated with the same highly conserved sequence motifs and that the coupled mechanism could support transport under ATP depletion conditions. In addition, evidence is provided that two other ABC exporters are also able to generate AMP and Pi upon addition of ATP, illustrating the general importance of our results. A contamination as source of the observed activity can be safely excluded especially based on the mutation data in Fig. 5 and Supplementary Fig. 1. Such a conclusion is also supported by the mutation data of LmrA^57^ and the high-temperature experiments on TmrAB (Fig. 7). Of course, our findings raise the immediate question of (1) whether a model could be proposed that accommodates all observations, (2) whether previously published biochemical data on MsbA agree with our model, (3) to which extent our proposal could be generalized and (4) whether any statement about its functional meaning could be made. All of these points will be discussed in the following.

**Figure 8:**
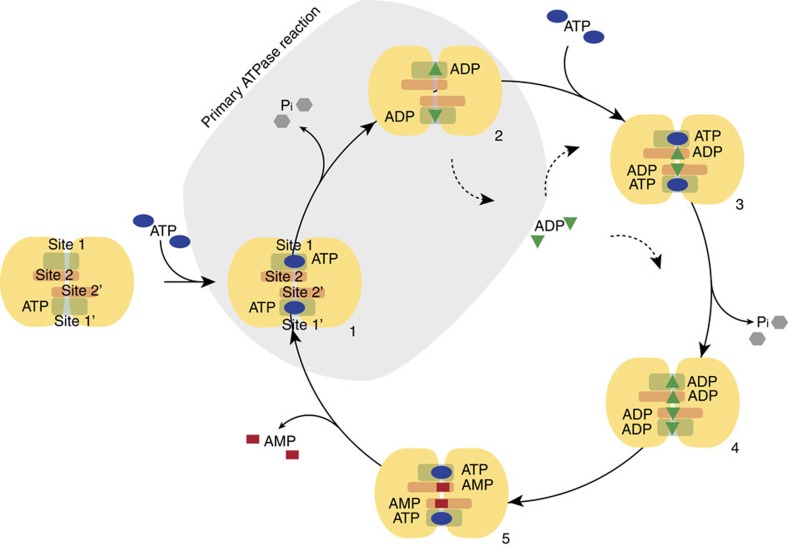
Proposed cycle for coupling ATPase with adenylate kinase activities in MsbA. Next to the canonical ATP-binding sites (site 1), two additional ADP-binding sites (site 2) are proposed involving Walker B and Q-loop of one and D-loop of the other NBD (Fig. 4a). This model allows suggesting a catalytic cycle with an ATP bound (1), ADP bound (2), ATP+ADP bound (3), ADP saturated (4) and finally ATP+AMP bound (5) state (see also Fig. 4 and Supplementary Fig. 7). The steps needed for the primary ATPase reaction as part of the normal catalytic cycle are highlighted.

The coupled ATPase-AK activity observed here requires a model that accommodates ATP-, ADP- and AMP-binding sites. Furthermore, it has to provide the possibility that two ADPs could bind in close proximity with respect to each other for the kinase reaction to take place. In the following discussion, we refer to the canonical ATP-binding sites between Walker A of one NBD and the signature motif of the other NBD as site 1 (Fig. 5a). The mutational studies described above have shown that alterations in Walker B, D-loop and Q-loop affect the coupled reaction. We therefore postulate that both parts of the coupled reaction are controlled by the same structural motifs. Indeed, the MsbA crystal structure (PDB: 3B60) shows that Walker B and Q-loop of NBD1 and D-loop of NBD2 seem to come in close proximity and could therefore potentially form a second binding site. Such an additional binding site would allow bringing two ADP molecules bound in both sites close enough to each other for the kinase reaction to take place. Interestingly, the crystal structure of the isolated NBD of *pf*SMC, a DNA repair enzyme with a topology related to ABC transporters, has been solved in complex with the kinase inhibitor Ap5A (PDB: 3KTA)^23^. This inhibitor mimics both ATP and AMP and occupies the canonical binding site as well as a second binding site that involves the highly conserved Q-loop glutamine. The potential location of both sites within the NBD of *pf*SMC is shown in Fig. 5a (top right) and compared with the same structural motifs in the NBD of MsbA (Fig. 5a, top middle). In both MsbA and *pf*SMC, the mutation of the Q-loop glutamine to alanine causes a strong reduction of the kinase reaction that could be explained by structural alterations of this second binding site. Such an additional binding site allows us to propose the hypothetical catalytic cycle illustrated in Fig. 8. Upon offering ATP to apo-MsbA, an ‘ATP bound state' forms in which sites 1 are occupied (**1**). ATP hydrolysis occurs resulting in an ADP-bound state (**2**) and in the release of Pi. These steps would correspond to the normal catalytic cycle of MsbA. Our real-time NMR data show that the amount of ADP increases in solution because of the ongoing hydrolysis reaction. Therefore, ADP must get released from MsbA before binding to sites 2 making free ADP available. Now, sites 1 can be occupied by another ATP molecule followed by ADP binding to sites 2 creating an ‘ATP-ADP state' (**3**). Alternatively, ADP binding to sites 2 could also take place after another hydrolysis step. In both cases, an ADP-saturated state will be formed (**4**). In this state, two ADPs are in close proximity with respect to each other in sites 1 and 2 so that the kinase reaction could take place. As a result ATP is formed at sites 1 and AMP at sites 2. This forms the ‘ATP+AMP bound state' (**5**). Furthermore, AMP should be released for the next cycle that starts with the hydrolysis of bound ATP. Such a model would be in agreement with our data that show no release of ATP during the catalytic cycle. The two postulated reaction centres, each formed by sites 1/2, result in uptake of ATP and release of Pi and AMP, but whether they both work sequentially or simultaneously cannot be concluded. The occurrence of an ATP-bound (**1**) and ADP-bound (**2**) state is well established but further evidence for additional nucleotide-bound states is provided by our nucleotide-binding experiments based on ^31^P-CP MAS NMR experiments. The data in Fig. 4 demonstrate simultaneous binding to of ADP·Vi and ADP-βS that resembles a trapped state (3), whereas spectra in Supplementary Fig. 2 demonstrate binding of ADP·Vi and AMP corresponding to a trapped state (5).

Although the hypothetical cycle proposed here is in line with our experimental data, it is also important to discuss how it relates to the ‘normal catalytic cycle' primarily based on ATP hydrolysis. Differences in nucleotide concentrations (ATP, ADP) can alter the rates of both reactions. Therefore, under high ATP levels, the ‘normal catalytic cycle' might dominate, whereas under low ATP and/or high ADP levels, the coupled cycle could come into play. This means that the AK reaction might not necessarily contribute to every ‘normal catalytic cycle' but that the protein might be able to adjust its activity because of altered environmental factors from catalysing primarily ATP hydrolysis to a coupled ATPase-AK cycle, for example, in cases of ATP depletion.

The coupled ATPase-AK mechanism observed here is a pronounced feature of the MsbA activity. It is well accessible by real-time solid-state NMR as all reactants can be observed simultaneously, while keeping MsbA within the lipid bilayer. However, as MsbA is a well-studied ABC exporter, the question arises of why this has not been described before or whether its surprising activity also surfaces by re-evaluating conventional ATPase activity assays used so far^30^,^34^,^58^. The ATPase activity of ABC transporters can be usually assessed by observing the generation of Pi or ADP through colorimetric or enzyme-linked assays, respectively. If an ABC transporter merely catalyses ATP hydrolysis, both assays should report the same activity. For MsbA, however, systematic differences are observed: the colorimetric Pi assay reports a specific activity of 9±1 μmol min^−1^ mg^−1^ for detergent solubilized MsbA, whereas the linked enzyme assay returns 300±50 nmol min^−1^ mg^−1^. These assay-dependent differences are consistent with the numbers reported in the literature for MsbA^30^,^31^,^33^,^59^ and can now be explained in the light of the coupled ATPase-AK mechanism: the colorimetric assay reports on the full coupled activity, whereas the linked enzyme assay compromises the kinase activity (see Supplementary Fig. 3, further discussion are provided as Supplementary Notes).

So far, substrate transport catalysed by ABC transporters has been shown to depend on ATP hydrolysis. Our data show that ATP hydrolysis can be coupled with an adenylate kinase mechanism under specific conditions. Whether any functional consequences can be derived from this observation needs to be therefore discussed. A cyclic coupling of an ATPase and a kinase reaction would result in a lower net consumption of ATP. The coupled process is energetically more favourable than ATP hydrolysis alone because the latter releases ΔG=−32.49 kJ mol^−1^, whereas the kinase reaction is almost a zero energy process corresponding to just ΔG=−0.30 kJ mol^−1^ (ref. 60). This means that the protein would be able to regenerate ATP partially without consuming anything more than its own by-product ADP. As a result, the basal ATPase activity observed for MsbA and other exporters might actually consume less ATP than assumed. More importantly, the possibility to couple ATP hydrolysis to an AK reaction would be of advantage in situations of ATP depletion that are quite common during the lifespan of an individual cell as recently demonstrated by single-cell *E. coli* experiments^61^. Under these conditions, ATP regeneration by the coupled ATPase-AK cycle would become important as MsbA might be able to protect cells against the toxic effects of lipid A accumulation^62^. A qualitative indication that transport indeed depends on the coupled cycle was obtained by utilizing the fluorescence properties in Hoechst-33342 (Fig. 6). Interestingly, ATP synthesis and substrate transport has been reported for LmrA under energy depletion conditions in an earlier study that could have been a result of the mechanism described here^63^.

A coupled ATPase-AK activity has been shown here for the ABC exporters MsbA, LmrA and TmrAB. The occurrence of an adenylate kinase activity along with ATP hydrolysis has also been described for three other ABC proteins, namely the CFTR^24^,^25^, an ATP-gated ion channel with ABC transporter-like topology including two NBDs, for the soluble DNA repair enzyme Rad50 and for the *pf*SMC^22^,^23^. So far, their adenylate kinase activity could not be generalized to ABC exporters. However, it has been demonstrated that the kinase activity is coupled to DNA repair in case of Rad50 (ref. 23) and to channel gating in case of CFTR^64^. These findings together with the transport data presented here demonstrate that the coupled mechanism could indeed be a general and relevant feature for proteins of the ABC family.

Our data show that the typical ABC exporter MsbA is able to catalyse both ATP hydrolysis and an adenylate kinase reaction that can be strongly coupled in a cyclic way. The AK reaction is most pronounced under conditions of low ATP and/or high ADP levels. Although ATP hydrolysis is driving the ‘normal catalytic cycle', a coupling to the AK mechanism could beneficially occur for example under situations of ATP depletion or other physiological stress conditions. Our additional demonstration on two more ABC transporters suggests these findings as a more general feature of prokaryotic ABC exporters. The highly conserved Walker A motif is important for both reactions and it assumes a very similar fold in most nucleotide-binding proteins from kinases, via regulators to ABC transporters. This suggests an evolutionary connection between these protein classes^41^,^42^.

## Methods

### Materials

Luria broth, Tris, HEPES and PIPES were obtained from Carl Roth GmbH (Karlsruhe, Germany). Ni-NTA beads were purchased from Qiagen (Hilden, Germany) and Biobeads were ordered from Bio-Rad Laboratories GmbH (München, Germany). ATP, ADP, ADP-βS, sodium meta-arsenite, phosphoenol pyruvate, lithium chloride and EDTA were purchased from Sigma-Aldrich GmbH (Schnelldorf, Germany). Complete protease inhibitor cocktail tablets, pyruvate kinase and lactate dehydrogenase were bought from Roche Diagnostics Deutschland GmbH (Mannheim, Germany) and Run Blue precast SDS–polyacrylamide gel electrophoresis gels from Expedeon Ltd (Cambridge, UK). Lipids were obtained from Avanti Polar Lipids, Inc. (Alabaster, AL, USA). All other compounds were from AppliChem GmbH (Darmstadt, Germany). For size-exclusion chromatography (SEC), Sephadex 200 10/300GL and PD10 column for buffer exchange were from GE Healthcare Europe GmbH (Freiburg, Germany). Primers for mutations were ordered from Eurofins Genomics GmbH (Ebersberg, Germany).

### Expression and purification

Expression and purification was essentially carried out as described before^38^. MsbA was cloned into a pET-19b vector containing an N-terminal His_10_-tag connected via an 11 amino acid peptide linker^65^. The plasmid was transformed into *E. coli C43(DE3)* cells for protein expression. For obtaining a high yield of MsbA, cells were grown initially in LB medium, collected by centrifugation and transferred to M9 minimal medium^66^. Expression was started by adding 10 ml of preculture to 1 litre of LB at 37 °C and 220 r.p.m. When the OD_600nm_ reached 0.5–0.6, cells were harvested and then resuspended into 500 ml of M9 minimal medium. The cells were further incubated at 37 °C, 220 r.p.m. for 1 h for adaptation. The protein expression was induced with 1 mM IPTG at 20 °C and 260 r.p.m. once the OD_600nm_ has reached a value between 1.7 and 2.1. After 17 h of expression, the cells were harvested and resuspended in lysis buffer (10 mM Tris, 250 mM Sucrose, 150 mM NaCl and 2.5 mM MgSO_4_, pH 7.5) with protease inhibitor, 0.5 mM dithiothreitol and DNAase. Membranes were prepared by passing the resuspended cells through a cell disrupter at a pressure of 1.5–1.7 kbar (2–3 times), followed by centrifugation at 4,500*g* (8,000 r.p.m., rotor F0850) for 10 min to remove cell debris followed by a final ultracentrifugation step at 223,000*g* (55,000 r.p.m., rotor 70 Ti) for 1 h. Membranes were solubilized in buffer B (50 mM HEPES, 300 mM NaCl, 5 mM MgCl_2_ and 10% Glycerol, pH 7.5) with 1.25% n-Dodecyl β-D-maltoside (DDM), 0.5 mM dithiothreitol and 10 mM imidazole at 4 °C overnight. The insoluble fraction was removed by ultracentrifugation at 223,000*g* (55,000 r.p.m., rotor 70 Ti) for 1 h. The remaining supernatant was loaded onto Ni-NTA prewashed with buffer B containing 50 mM imidazole. After 1.5–2 h of binding, elution was carried out using buffer B containing 0.015% w/v DDM and 400 mM imidazole. SDS–polyacrylamide gel electrophoresis, SEC using a Sephadex 200 column and MALDI-MS confirmed high purity and homogeneity of the protein.

### Reconstitution

A lipid mixture of DMPC/DMPA (9:1) at molar lipid to protein ratios of 75:1 was used for reconstitution. Lipids were solubilized in CHCl_3_/CH_3_OH (2:1) and dried under a stream of nitrogen gas followed by vacuum rotor evaporation. The dried lipids were resuspended in buffer (50 mM HEPES and 50 mM NaCl, pH 7) and extruded 5 times through membranes with pore diameters of 0.2 μm and 8 times with pore diameters of 0.1 μm. Liposomes were destabilized with 3 mM DDM^38^. After drop-wise addition of protein in the DDM/liposome solution, the mixture was incubated at room temperature for 30 min. Detergent was removed by biobeads (80 mg ml^−1^) that were first applied overnight at 4 °C and then again for 1 h at room temperature. The reconstituted protein was pelleted at 58,000*g* (28,000 r.p.m., rotor 70 Ti) for 25 min. The supernatant buffer was decanted carefully. The pellet was then resuspended in 700 μl of 20 mM HEPES. The pelleting and resuspension procedure was repeated three times. This process ensures that soluble components are removed. MsbA shows the same ATPase activity in DMPC/DMPA compared with *E. coli* lipids under similar reconstitution conditions^38^. The use of *E. coli* lipid mixtures was however avoided because of residual amounts of lipid A, the native substrate of MsbA.

### MsbA mutants

Single-point mutations in MsbA (K382A, Q424A, S481A, D505A and D511A) were introduced into the plasmid using the following primers: (1) Q424A Fwd: 5′-GTTAAACAGATGGACATTCGCCGACACCAGAGCAACCTGG-3′ and Rev: 5′-CCAGGTTGCTCTGGTGTCGGCGAATGTCCATCT GTTTAAC-3′; (2) K382A Fwd: 5′-GCTGGCGATGGTTGATGCACCCGAACCAGAGCGT-3′ and Rev: 5′-ACGCTCTGGTTCGGGTGCATCAACCATCGCCAGC-3′; (3) D505A Fwd: 5′-GCCGAGGTAGCTTCGGCCAGAATCAGAATCG-3′ and Rev: 5′-CGATTCTGATTCTGGCCGAAGCTACCTCGGC-3′; (4) D512A Fwd: 5′-CGTTCGGATTCGGTAGCCAGAGCCGAGGTAG-3′ and Rev: 5′-CTACCTCGGCTCTGGCTACCGAATCCGAACG-3′; and (5) S481A Fwd: 5′-GCTGACCGCCAGCGAGCAGCACGCC-3 and Rev: 5′-GGCGTGCTGCTCGCTGGCGGTCAGC-3′. The PCR conditions for each mutant were optimized and mutations were confirmed by sequencing (SRD Biotech GmbH). All mutants were expressed, purified and reconstituted in the same way as wild-type MsbA as described above. The same amount of protein (8 mg) was used in each case for reconstitution and NMR experiments. The basal ATPase activity of wild type and mutants was determined using a colorimetric assay (see Supplementary Information for further details). The activity (μmol min^−1^ mg^−1^) in 0.015% DDM was 9.0±0.5 (wild type), 0.6±0.1 (K382A) and 9±1 (Q424A). For S482A, D505A and D512A, almost no activity could be measured. All NMR experiments on these mutants were carried out as described for the wild type.

### Hoechst-33342 transport assay

*Preparation of ISOVs.* Wild-type MsbA, MsbA K382A and empty vector (pET 19b) were transformed in C43DE3 (ΔAcrB) cells (kindly provided by Professor Dr Martin Pos, Frankfurt). A preculture from freshly transformed cells was used for the overexpression of MsbA. The expression was started in LB at 37 °C, 220 r.p.m. and induced at an OD_600_ of 0.5–0.6 for 3 h at 260 r.p.m. The cells were harvested and frozen at −80 °C overnight. The cell pellet was then resuspended in the lysis buffer and passed through the cell disruptor three times at a pressure of 1.7 kbar. The solution after cell disruption was centrifuged at 5,500*g* for 15 min to remove whole cells and cells debris. ISOVs were then obtained by a final ultracentrifugation step at 223,000*g* for 1 h. The ISOV pellet was resuspended in 50 mM potassium phosphate buffer, pH 7.2, 10% v/v glycerol for the assay^54^,^67^,^68^.

*Fluorescence spectroscopy*. ISOVs were diluted to a final membrane protein concentration of 0.2 mg ml^−1^ as determined by a Lowry assay. Fluorescence decay as a result of H33342 transport inside the vesicles was followed using an excitation wavelength of 355 nm and an emission wavelength of 457 nm with a slit width of 5 and 3 nm, respectively. The ISOVs were incubated with 6 mM MgSO_4_ until the fluorescence stabilized. Furthermore, H33342 equivalent to a final concentration of 6 μM was added followed by addition of nucleotide with subsequent recording of fluorescence intensity time courses. The experiment was carried out with different concentrations of nucleotides, that is, 2, 3 and 5 mM for ATP, ADP and AMP, respectively.

### ^31^P-MAS NMR

The MsbA proteoliposomes pellet was transferred into a 4 mm MAS rotor (50 μl) by centrifugation and kept on ice. An amount of 20 μl 100 mM MgATP solution in 20 mM HEPES pH 7 was added to the pellet in the rotor so that a molar ATP/MsbA ratio of 16 was achieved. An ATP/Mg^2+^ ratio of 10:3 was used that is the best optimum with respect to ^31^P line width and activity (Supplementary Fig. 4). After closing, the MAS rotor was quickly transferred to a Bruker Avance 600 WB spectrometer equipped with a HXY-MAS probe tuned to ^31^P and ^1^H. The ^1^H–^31^P Larmor frequencies were 600.13 and 242.93 MHz, respectively. The probe was pre-equilibrated at 270 K. The sample spin rate was adjusted to 10 kHz. The detection of the MsbA catalysed turnover of nucleotide by direct ^31^P polarization started immediately after sample spinning stabilized. All experiments were carried out at 270 K nominal probe temperature in order to slow down the reaction so that a good signal-to-noise ratio per unit time and for sampling the time evolution of the ongoing reaction with enough data points could be achieved.

^31^P MAS NMR spectra were recorded using direct polarization with a 4 μs 90° pulse and 62.5 kHz ^1^H SPINAL-decoupling^69^ during 30 ms acquisition with a recycle delay of 3 s. All direct polarization spectra were acquired at 270 K with 10 kHz sample spinning, unless stated otherwise. All spectra were recorded using 512 scans that correspond to a time difference of 25.06 min between each point. The experimental dead time between adding ATP to the proteoliposomes and starting the NMR data acquisition was ∼5 min. The time-resolved spectra were stored into a serial FID in order to simplify processing and data analysis. Spectra were processed within TopSpin 2.1 using 16 k zero-filling and 20 Hz exponential line broadening before Fourier transformation. Chemical shift referencing was carried out indirectly with respect to 10% phosphoric acid at 0 p.p.m. via crystalline triethylphosphine set to 58.62 p.p.m. Time-resolved pseudo-two-dimensional data sets were subjected to a complete peak deconvolution using the mdcon routine built into TopSpin 2.1 (Bruker, Karlsruhe). Chemical shifts, line widths, intensities and line shapes (Lorentzian/Gaussian) were fitted simultaneously to each resonance in each time slice. All samples contained comparable amounts of protein (within the error limits of the Lowry assay) and lipids (as verified by the intensity of the ^31^P lipid resonance). The lipid resonance therefore served as internal normalization constant for all ^31^P progress curves.

The experiments with ADP-βS (Fig. 2) were carried out similarly but at 290 K and with a molar ATP/MsbA ratio of 12. The spectra in Fig. 3c,d were recorded using a molar ratio of MsbA/ATP/ADP/AMP=1:16:320.

Trapping of reconstituted MsbA with ADP.Vi (Fig. 4) was done essentially as described before^30^. Briefly, the reaction mixture contained reconstituted protein (8 mg) in 50 mM HEPES, 10 mM ATP, 10 mM MgCl_2_ and 3 mM orthovanadate solution. This solution was subjected to freeze–thaw cycles in order to improve the MsbA accessibility in the proteoliposome sample followed by incubation at 37 °C for 20 min. Afterwards, the sample was pelleted to remove excess reagents and resuspended in 20 mM HEPES. Subsequently, ADP-βS was added to these samples in excess. All further preparation steps and experimental conditions were as described above. A CP contact time of 4.5 ms was used. For each trapped state, 4,096 scans at a nominal probe temperature of 260 K with a recycle delay time of 3 s were accumulated.

### Verifying experimental conditions used for ^31^P-MAS NMR

Because of the limited detection sensitivity of NMR spectroscopy, conditions had to be selected under which the reaction runs slowly enough so that sufficient signal for each time step could be accumulated. This has been achieved by lowering the reaction temperature to 270 K. At higher temperature, the reaction takes place as expected but at accelerated rate and spectra are less well resolved (Supplementary Fig. 5). All experiments were carried out with MsbA reconstituted in DMPC/DMPA (see above). The effect of the membrane environment has been tested by control experiments in which MsbA was solubilized in DDM (Supplementary Fig. 6), reconstituted into *E. coli* lipids (Supplementary Fig. 7) and within crude *E. coli* membranes (Supplementary Fig. 8). In all of these cases, MsbA-dependent formation of AMP has been observed. All experiments were carried out with excess ATP and without explicit addition of substrate. MsbA-dependent AMP formation is also observed under ATP-saturation conditions (Supplementary Fig. 9) and in the presence of substrate Hoechst 33342 (Supplementary Fig. 10).

### LmrA and TmrAB

For the spectra in Fig. 7a, LmrA was overexpressed in *E. coli* as previously described^54^. Briefly, the plasmid was transformed in C43(DE3) cells and expressed with the spin-down protocol as described above for MsbA. The protein was then purified in 20 mM HEPES containing 0.05% DDM and 150 mM imidazole. Then, 8 mg protein was reconstituted at a molar LPR of 250:1 into DMPC/DMPA (9:1) liposomes. Spectra were recorded under conditions identical to those described above for MsbA, that is, a ATP/LmrA ratio of 16 mol mol^−1^ was used. The TmrAB sample for the data in Fig. 7c was prepared as described before^55^. The protein was expressed in *E. coli* BL21(DE3). Membranes were pelleted by centrifugation of lysed cells at 120,000*g* for 1 h and solubilized with solubilization buffer (20 mM HEPES, 300 mM NaCl, pH 7.5, and 20 mM β-DDM) by incubation for 1 h at 20 °C. Detergent-solubilized His-tagged TmrAB was bound to Ni-NTA agarose (Qiagen) in the presence of 30 mM imidazole (pH 8.0) and washed with purification buffer containing 50 mM imidazole and eluted with purification buffer supplemented with 300 mM imidazole. Finally, TmrAB was purified via SEC using a TSK G3000SW column (Tosoh Bioscience LLC) in SEC buffer (20 mM HEPES, 150 mM NaCl, pH 7.2, and 1 mM β-DDM). Solution state NMR spectra were recorded at 600 MHz (Bruker Avance 600, 5 mm cryoprobe) and 341 K with 256 scans for each time step. The sample contained 15.5 μM TmrAB (0.05% DDM) and 250 μM MgATP (Mg^2+^/ATP=3:10).

### Data availability

The authors declare that all data supporting the findings of this study are available within the article, its Supplementary Information file and from the corresponding author on reasonable request. The following PDB structures were used in this study: 3B60, MsbA-AMP-PNP and 3KTA, isolated NBD of *pf*SMC complex with the kinase inhibitor Ap5A.

## Additional information

**How to cite this article:** Kaur, H. *et al*. Coupled ATPase-adenylate kinase activity in ABC transporters. *Nat. Commun.* **7**: 13864 doi: 10.1038/ncomms13864 (2016).

**Publisher's note**: Springer Nature remains neutral with regard to jurisdictional claims in published maps and institutional affiliations.

## Supplementary Material

Supplementary Information Supplementary Figures, Supplementary Notes, Supplementary References.

## Figures and Tables

**Table 1:** MsbA single-point mutations used for probing its catalytic centre.

**Motif**		**Mutation**
Walker A (WA)	376-GRSGSG**K**ST-384	K382A
Q loop (Q)	422-VS**Q**NV-426	Q424A
Signature motif (S)	481-L**S**GGQ-485	S482A
Walker B (WB)	501-ILIL**D**E-506	D505A
D loop (D)	509-SAL**D**-512	D512A

The bold entries indicate point of mutation.
