# Supplementary material for: Coupled ATPase-adenylate kinase activity in ABC transporters
Source: Nat Commun. 2016 Dec 22;7:13864. doi: 10.1038/ncomms13864 (PMC5192220; doi:10.1038/ncomms13864)
Supplement: Supplementary Information — Supplementary Figures, Supplementary Notes, Supplementary References. [file ncomms13864-s1.pdf]

# Supplementary Information

## Supplementary Figures

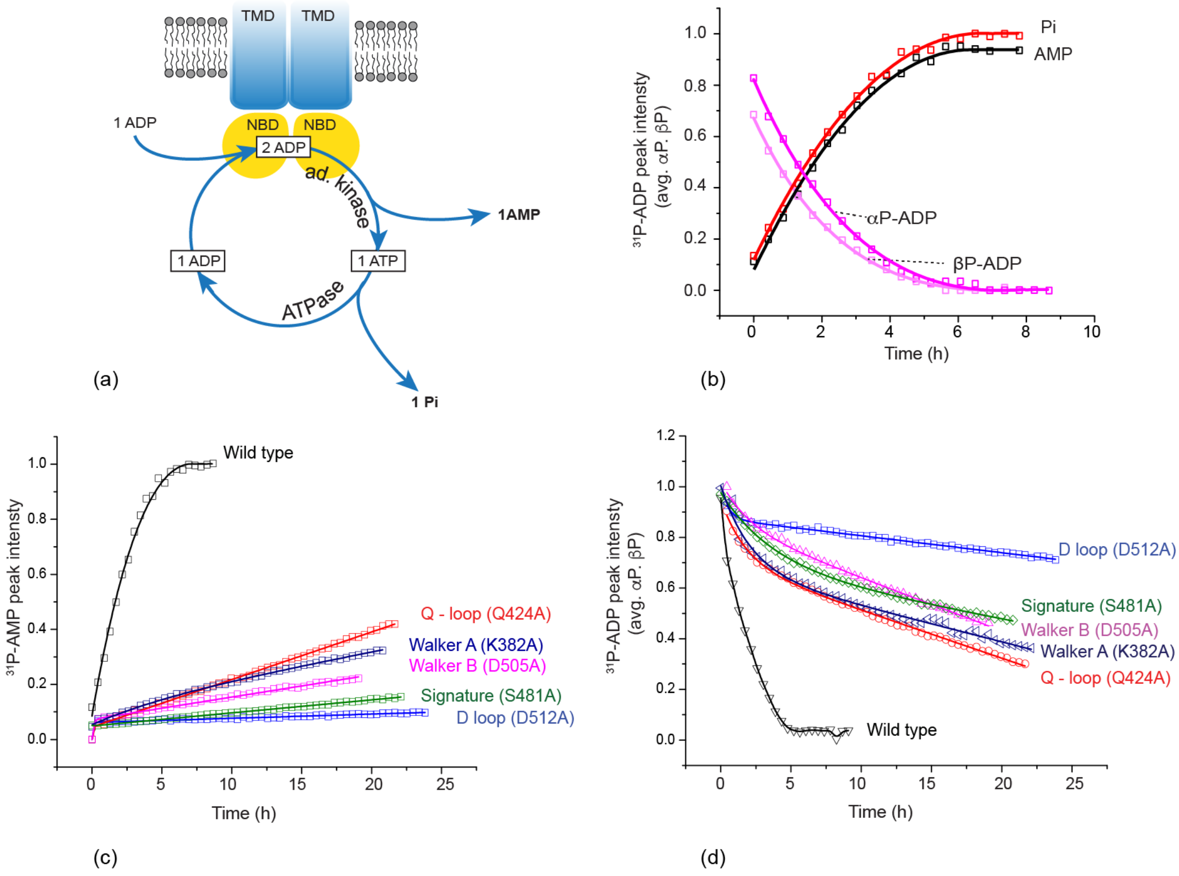


**Supplementary Figure 1:** Mutational analysis of the ADP-based coupled ATPase-AK activity. (a) Proposed model for the coupled ATPase/AK reaction upon addition of MgADP to MsbA. A reverse adenylate kinase reaction turns ADP into AMP and ATP. Subsequently, ATP is consumed by MsbA and turned into ADP and Pi. (b) $^{31}$P progress curves obtained from integral peak intensities from the deconvoluted data set in Fig. 1c shows an approximate stoichiometry of initial ADP to final AMP and Pi of 1:1:1, which is in agreement with the proposed cycle in (a). (c) Mutational analysis of the coupled ATPase/kinase activity of MsbA in the presence of MgADP. All single point mutations are summarized in Fig. 5. (a) Progress curves of the $^{31}$P-AMP peak intensities demonstrate much reduced generation of AMP by the mutants as compared to wild-type. (d) Progress curves of the $^{31}$P-ADP peak intensities follow the trend observable by the addition of MgATP as depicted in Fig. 5. The D512A mutation causes the slowest ADP consumption corresponding to the observation in Fig. 5. The experimental conditions were as described for Fig. 5, but MgATP was replaced by MgADP.

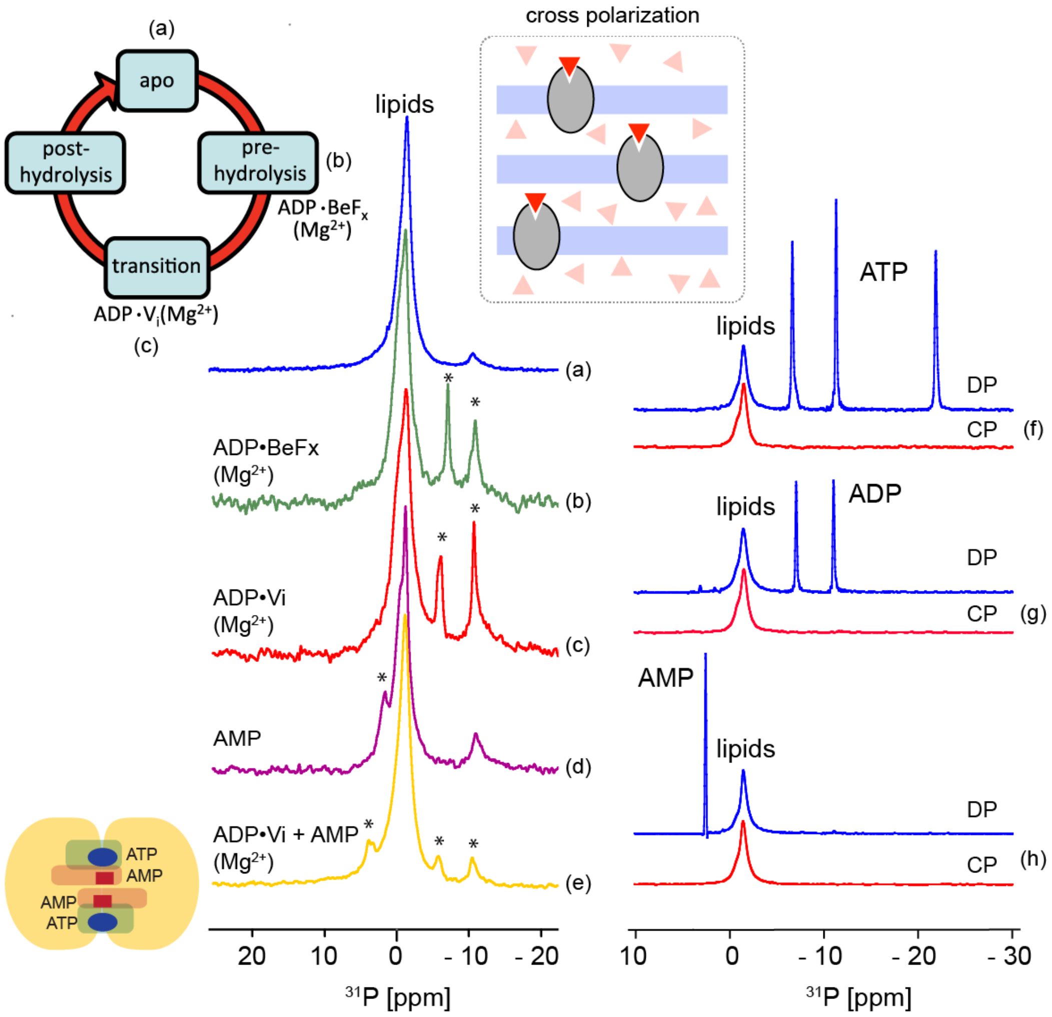


**Supplementary Figure 2:** Cross-polarized (CP) $^{31}$P-MAS NMR spectra of MsbA proteoliposomes in different nucleotide bound states. The dipolar-based CP polarization transfer from the homogeneous $^{1}$H network to $^{31}$P can be used to discriminate bound, immobile (red triangles) from non-bound nucleotides (pink triangles) in solution (for a review see [1]). The later will not cross polarize and do not appear in the spectra. (a) Apo-state MsbA. (b) MsbA trapped in a pre-hydrolysis state. Two resonances from $\alpha$P and $\beta$P of bound ADP can be detected. (c) MsbA in the Vi-trapped transition state. (d) Spectrum of apo-state MsbA after addition of AMP. Under CP, a signal of bound AMP is observed. (e) Spectrum of Vi-trapped MsbA recorded after addition of AMP. Signals for simultaneously bound ADP•Vi and bound AMP are observed. This spectrum provides an indication for the population of an ATP-AMP-bound state of MsbA as postulated in Fig. 8. The small chemical shift change of AMP between (d) and (e) could be due to trapping by ADP•Vi. Trapping of MsbA was done essentially as described before.[2] (f-h) Control experiments comparing CP and direct polarized $^{31}$P-MAS NMR spectra of ATP, ADP and AMP in the presence of DMPC:DMPA (9:1) liposomes. Unlike in the presence of MsbA, no CP signal can be detected, which verifies that the nucleotides do not bind unspecifically to lipids. The liposomes were prepared as described for proteoliposomes except that elution buffer without protein was used as the first step of reconstitution.

Spectra were recorded under the same conditions as described in Materials and Methods. A CP contact time of 4.5 ms was used. For each trapped state, 4096 scans at a nominal probe temperature of 260 K with a recycle delay time of 3 sec were accumulated.

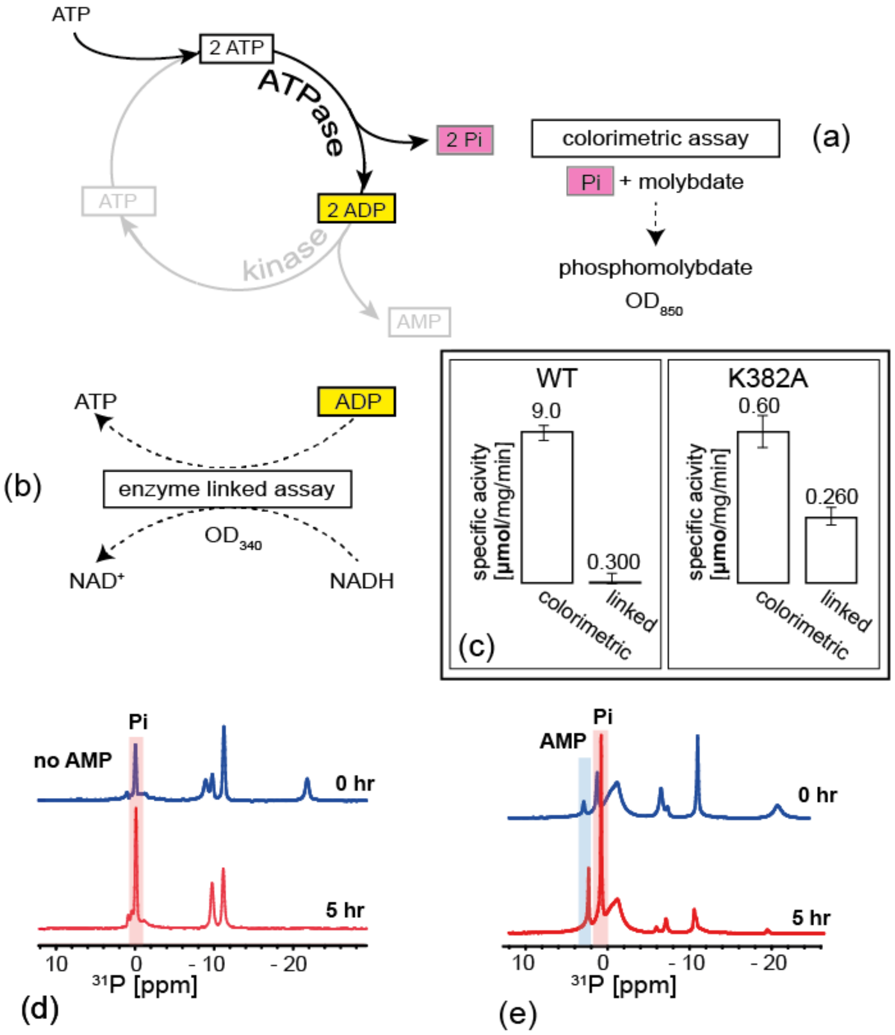


**Supplementary Figure 3:** Probing the basal ATPase activity of MsbA using conventional biochemical assays. (a) Colorimetric assay: $P_i$ released as a result of ATP hydrolysis forms a blue coloured complex with molybdate which is quantified by measuring OD 850 nm. (b) Enzyme linked assay: ADP produced as a result of ATP hydrolysis is consumed by pyruvate kinase leading through a cascade of coupled reactions in the presence of PEP and Lactate Dehydrogenase to the conversion of NADH to $NAD^+$ detected at OD 340 nm. (c) Comparison of results from both the assays with WT and Walker A (K382A) mutant. (d) $^{31}$P MAS NMR proves that under conditions of the enzyme linked assay no AMP is produced. The kinase reaction of MsbA is suppressed due to excess pyruvate kinase in the reaction mixture. (e) $^{31}$P MAS NMR shows that under the conditions of the colorimetric assay, the coupled ATPase/kinase reaction takes place. The data are further discussed in the Suppl. Notes section.

Experimental details for the colorimetric assay: The reaction mixture containing MsbA (0.015% DDM) and ATP (5 mM) in buffer (50 mM HEPES, 50 mM NaCl, 10 mM $MgCl_2$) was incubated at 37˚ C for 20 min. Control samples for each reaction were kept on ice. The reaction was stopped by adding 12 % SDS. The release of inorganic phosphate ($P_i$) was measured using the molybdenum blue method [3,4].

Experimental details for the enzyme-linked assay: The reaction mixture (25 mM PIPES pH 6.8, 50 mM LiCl, 0.1 mM EDTA, 15 mM MgCl2, 1 mM Phosphoenol Pyruvate, 0.5 mM NADH) with PK/LDH 5.8 U was incubated at 30° C for 5 min before adding ATP (5 mM) and MsbA (1 µg). This coupled reaction leads to decay of NADH to NAD+, which can be monitored as time course at OD 340 nm.[5]

Experiments were repeated three times and the activity was calculated as the mean value. Error bars correspond to standard deviations.

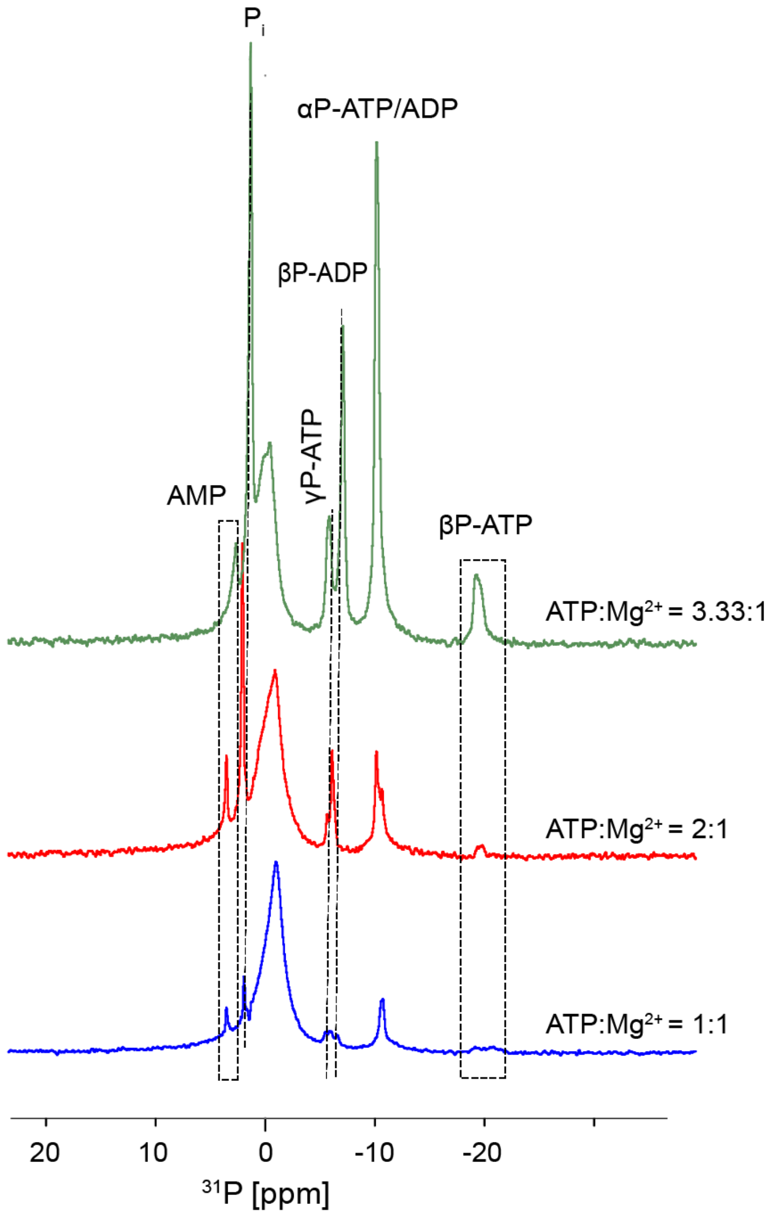


**Supplementary Figure 4:** Optimization of the ATP: $Mg^{2+}$ ratio. Choosing the correct stoichiometry between ATP and $Mg^{2+}$ is crucial to find an optimum between $^{31}P$ line width and protein activity, since $Mg^{2+}$ is needed for activity, but complex formation can result in extreme exchange broadening of the spectral lines. Here, ratios of 1:1, 2:1 and 3.33:1 have been tested. Shown are $^{31}P$-MAS NMR spectra of MsbA proteoliposomes recorded at the beginning of the real-time detection as shown in Fig. 1. Best resolved spectra were obtained for 3.33:1, which was subsequently used throughout this study. Spectra were recorded as described under Materials and Methods. The spectra do not correspond to exactly the same time points.

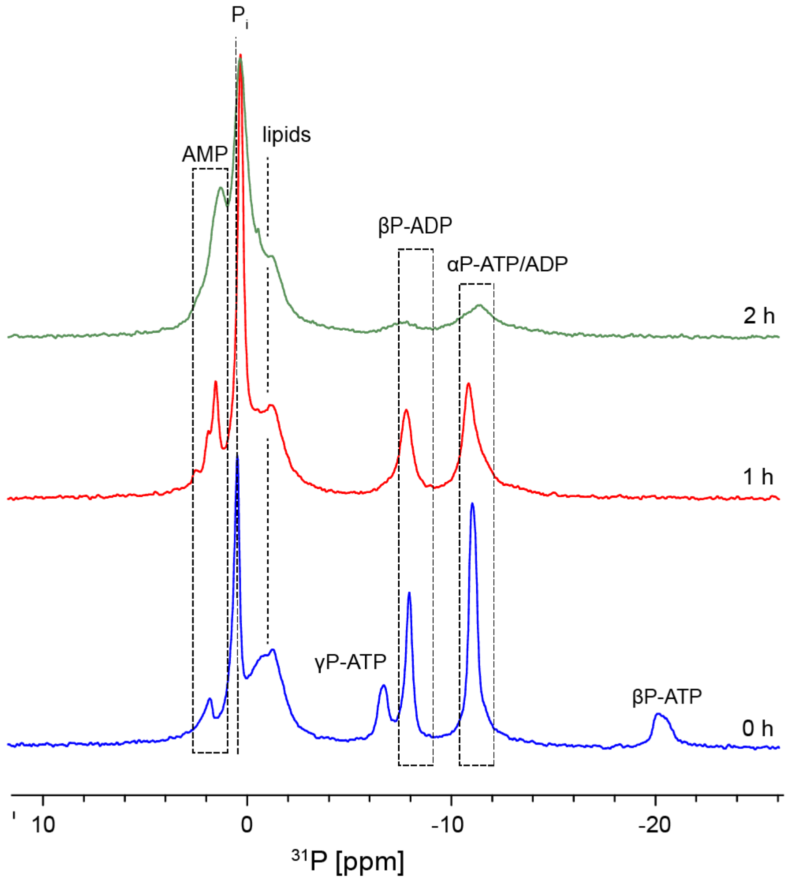


**Supplementary Figure 5:** The coupled ATPase/AK reaction of MsbA at 290 K. Most experiments described in this paper were recorded at 270 K. In order to exclude temperature-induced artifacts, a control experiment was carried out at 290 K. Also under these conditions, generation of AMP and Pi indicative for the coupled reactions was observed. The reaction runs faster and lines broaden significantly. The MsbA proteoliposomes were prepared as described for all other experiments.

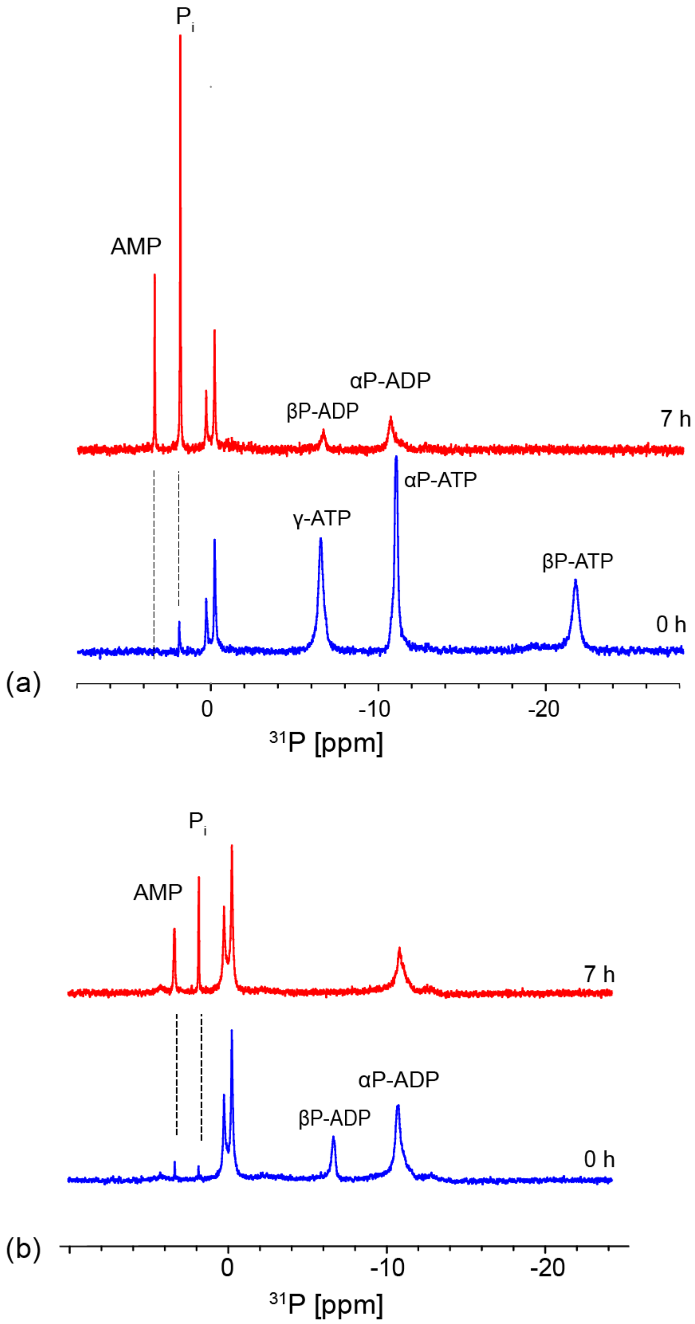


**Supplementary Figure 6:** $^{31}$P solution-state NMR of MsbA in DDM in the presence of ATP and ADP. In order to test whether the coupled ATPase/AK activity can also be observed in MsbA solubilized in DDM, solution state NMR spectra were recorded upon addition of ATP (a) and ADP (b). In both cases, formation of AMP is observed as detected for proteoliposomes (Fig. 1). The experiment was carried out with 0.125 mM MsbA in 0.5% DDM. ATP/ADP (100 mM) were allowed to react at room temperature. Spectra were recorded on Bruker Avance 600 spectrometer using a 5mm cryo-probe.

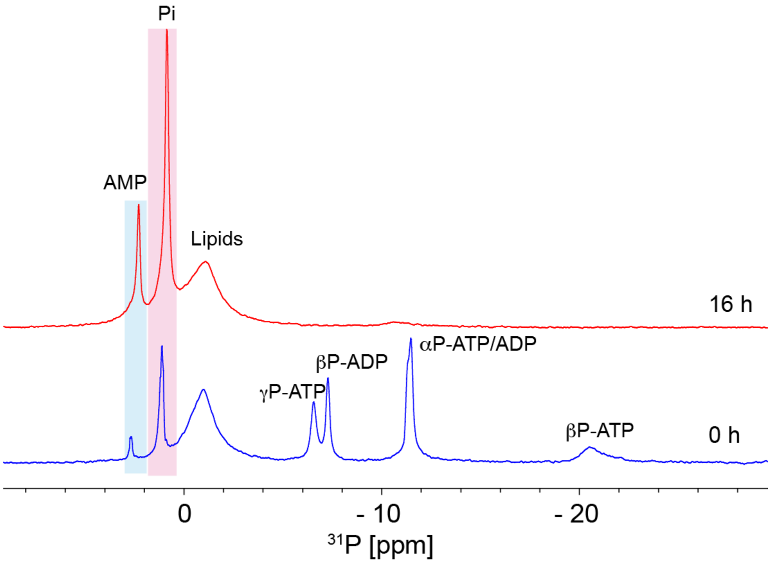


**Supplementary Figure 7:** $^{31}$P-MAS NMR on MsbA in reconstituted into *E. coli* lipids. Upon addition of MgATP, the generation of Pi and AMP at a final stoichiometry of 2:1 is observed. The same experimental conditions as described above were used. Reconstitution of MsbA (6 mg) in *E. coli* polar lipids at a molar lipid to protein ratio of 75:1 was carried out as described before[6].

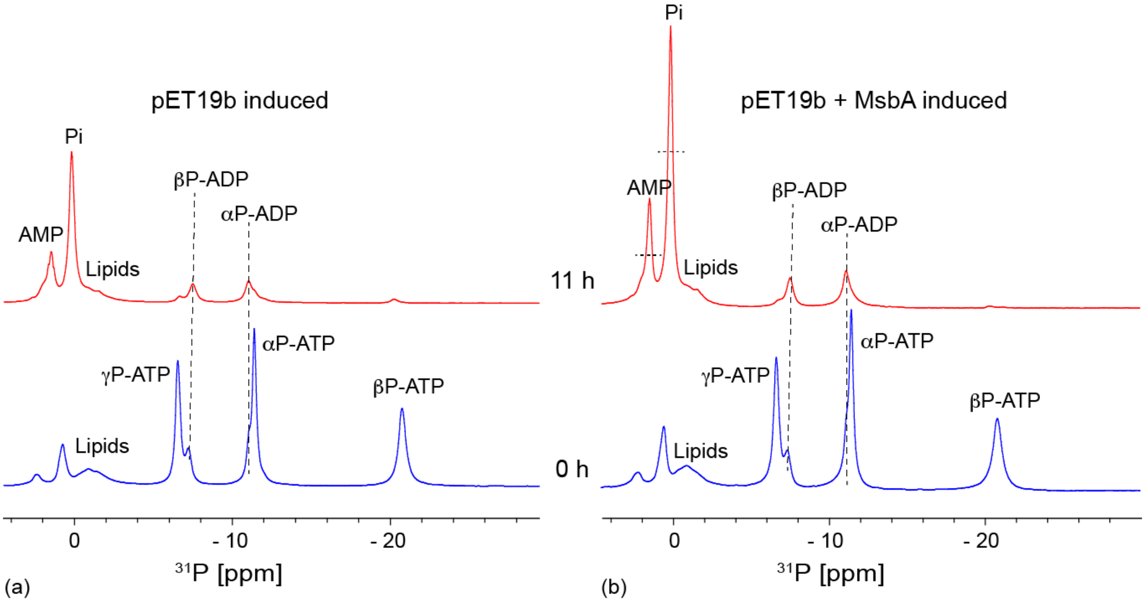


**Supplementary Figure 8:** $^{31}$P-MAS NMR on MsbA in crude *E. coli* membrane samples. To visualize activity of MsbA in native environment – membrane fractions from an expression with MsbA and empty vector (control) were prepared. These crude membranes were weighed to 30 mg and ATPMg was added to observe the coupled reaction at 270 K. (a) crude *E. coli* membranes induced with empty vector show much less AMP and Pi phosphorus formed as compared to (b) *E. coli* membranes induced with vector containing MsbA in the same time. The background activity in (a) can be explained by the occurrence of native ABC transporters and other membrane proteins. A pronounced activity due to the presence of overexpressed MsbA in the membranes is observed in (b).

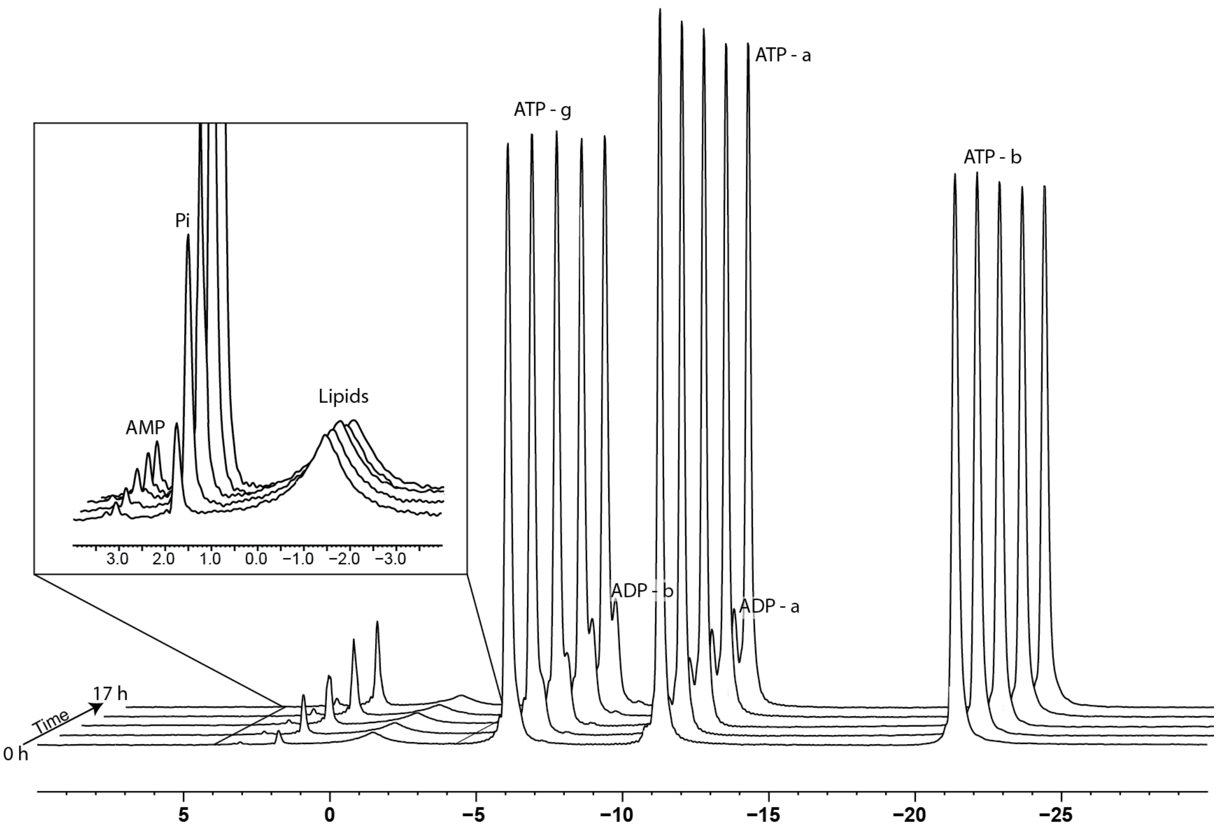


**Supplementary Figure 9:** The coupled ATPase/AK activity of MsbA in excess ATP: 1mg MsbA with excess ATP.Mg observed with real time $^{31}$P-NMR at 270 K. Over 17 h of measurement time, ATP concentration apparently remains constant. Under these excess conditions, generation of AMP and Pi from the starting point is indicative of the intrinsically coupled reaction. The MsbA proteoliposomes were prepared as described for all other experiments.

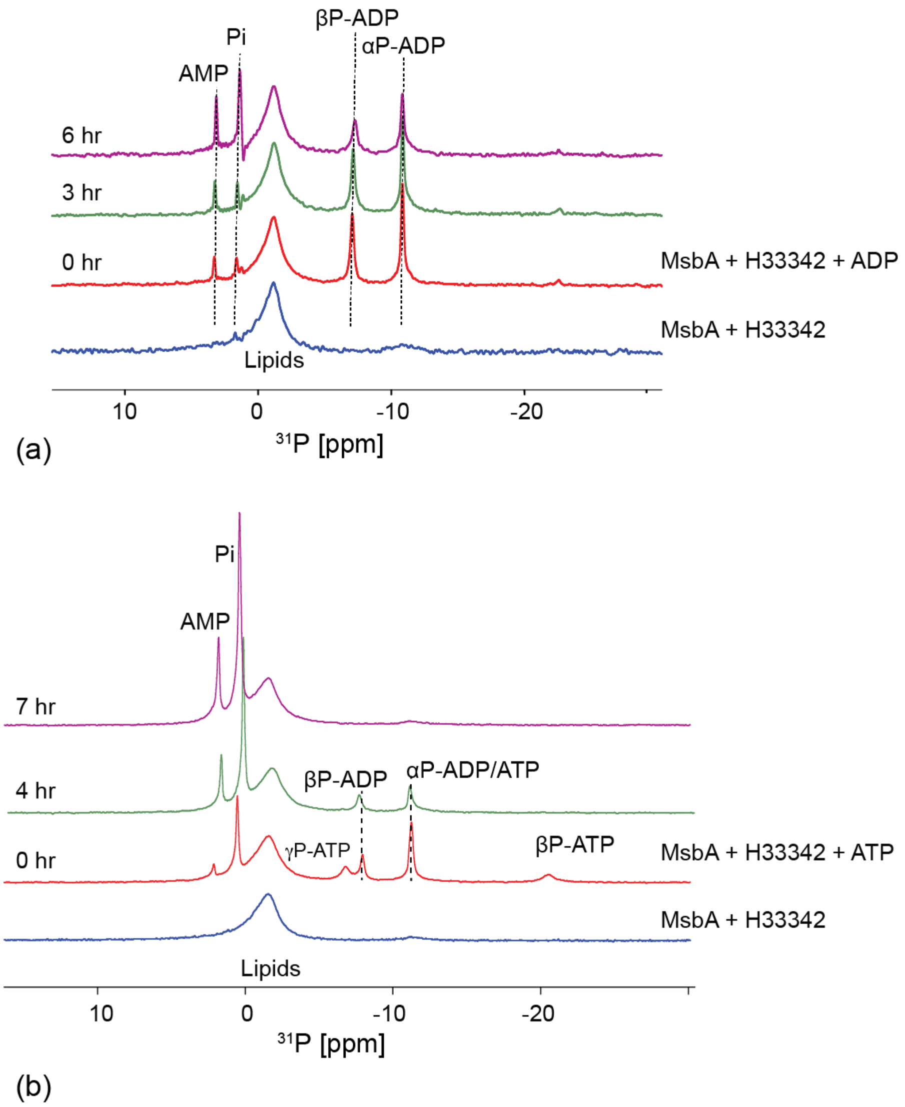


**Supplementary Figure 10:** Coupled ATPase/kinase activity in the presence of the substrate Hoechst-33342. (a) MgADP was added to MsbA proteoliposomes sample containing the substrate Hoechst-33342. As observed without substrate (Fig. 1c), ADP is turned over into AMP and Pi. The experiment was carried out under identical conditions as described before. The molar ratio MsbA:ADP was 1:16 and ADP:$Mg^{2+}$ 10:3. Hoechst-33342 was added at MsbA : H33342 = 1:8 mol/mol. (b) The same experiment as in (a) but in the presence of MgATP.

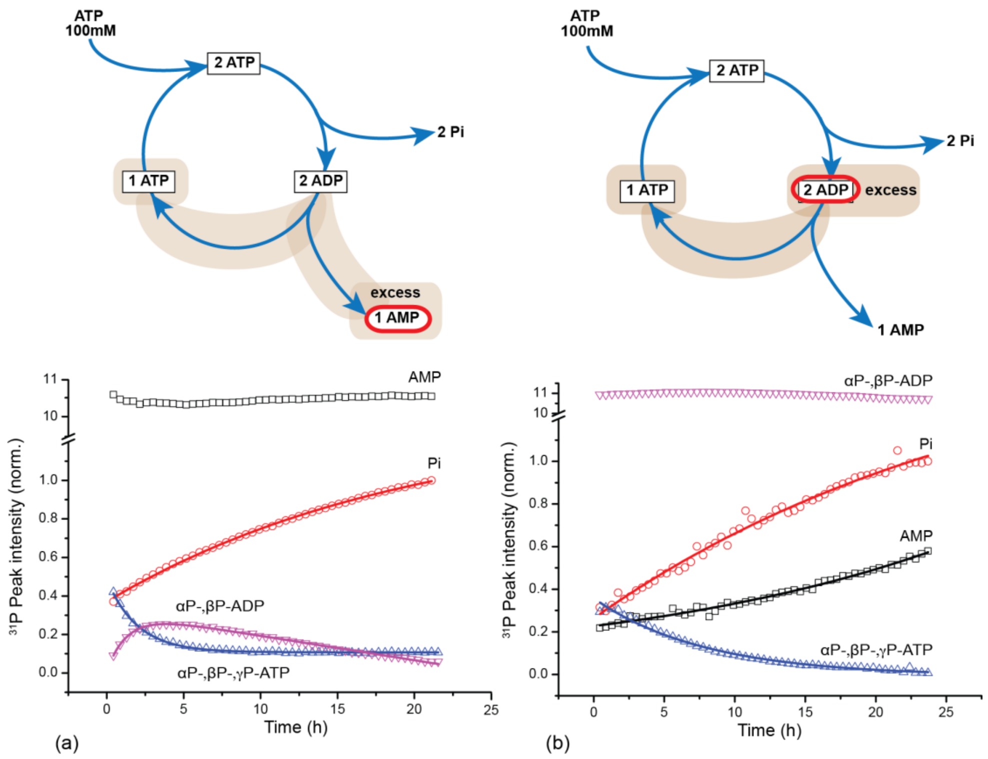


**Supplementary Figure 11:** Fully deconvoluted progress curves for the data sets shown in Figs. 3c and 3d recorded under conditions of excess AMP (a) and excess ADP (b). A stoichiometry of MsbA:ATP:ADP/AMP of 1:16:320 was used. Time-resolved spectra were recorded at 270 K with 512 scans for each increment.

## Supplementary Notes

### The coupled ATPase/kinase activity probed by biochemical ATPase assays

MsbA shows both an ATPase as well as a kinase activity. This phenomenon is well accessible by real-time solid state NMR since all reactants can be observed simultaneously, while keeping MsbA within the lipid bilayer. However, since MsbA is a well-studied ABC exporter, the question arises whether this surprising activity also surfaces by re-evaluating previously published data. The stoichiometric ratio of initial ATP to final Pi would be a good indicator for assessing whether the primary ATP hydrolysis reaction is the sole mechanism underlying the catalytic activity (see Fig. 3b), but to the best of our knowledge, such values have not been reported for full-length exporters. Instead, conventional ATPase activity assays have been used so far [2,7,8] and systematic differences have been reported reflecting indirectly the coupled ATPase/AK cycle.

The activity of MsbA has been assessed by either observing the generation of $P_i$ through a colorimetric assay based on complex formation of $P_i$ with ammonium molybdate [4] (Suppl. Fig. 3a) or by observing the generation of ADP. The latter is monitored through an enzyme-linked assay in which pyruvate kinase turns ADP and phosphoenol pyruvate into ATP and pyruvate. The latter is then converted by lactate dehydrogenase into lactate under consumption of NADH, which is monitored optically at 340 nm (Suppl. Fig. 3b) [5].

If an ABC transporter merely catalyzes ATP hydrolysis, both assays should report the same activity. However, significant differences are observed: The colorimetric Pi assay reports a specific activity of 9±1 µmol/min/mg for detergent solubilized MsbA, while the linked enzyme assay returns 300±50 nmol/min/mg (Suppl. Fig. 3c). These systematic assay-dependent differences are consistent with the numbers reported in the literature for MsbA: Activities in the range of µmol/min/mg have been reported using the colorimetric assay [2,9], while enzyme-linked assays returned systematically smaller values in the range of nmol/min/mg [10,11]. They can be explained by the coupled ATPase/kinase mechanism occurring in MsbA. In the linked enzyme assay (Suppl. Fig. 3b), initial ADP generated by the ATPase activity of MsbA is consumed by the pyruvate kinase, which is in excess. As a consequence, the kinase reaction of MsbA is compromised and a lower overall activity is observed. As further evidence, we have recorded $^{31}$P-MAS NMR spectra of the reaction mixtures used for the linked enzyme assay. The spectra show clearly, that indeed no AMP is produced and therefore the kinase activity of MsbA cannot be detected (Suppl. Fig. 3d). In contrast, the colorimetric assay does not interfere with the ongoing protein activity. $^{31}$P MAS NMR spectra of the corresponding reaction mixture show full ATPase and kinase activity with final production of AMP and Pi (Suppl. Fig. 3e).

The differential response of both assays to both activities is also evident from data obtained for the K382A (Walker A) mutant of MsbA, which affects mainly the kinase and ATPase activity of MsbA (Fig. 5). Indeed, in contrast to wild type MsbA, the specific activities determined by both assays are almost the same, since the kinase activity is much reduced (Suppl. Fig. 3c).

## Supplementary References


1. Lakatos, A., Mors, K. & Glaubitz, C. How to investigate interactions between membrane proteins and ligands by solid-state NMR. *Methods Mol Biol* **914**, 65-86 (2012).
2. Eckford, P.D. & Sharom, F.J. Functional characterization of Escherichia coli MsbA: interaction with nucleotides and substrates. *J. Biol. Chem.* **283**, 12840-50 (2008).
3. Chifflet, S., Torriglia, A., Chiesa, R. & Tolosa, S. A method for the determination of inorganic phosphate in the presence of labile organic phosphate and high concentrations of protein: application to lens ATPases. *Anal. Biochem.* **168**, 1-4 (1988).
4. Gonzalez-Romo, P., Sanchez-Nieto, S. & Gavilanes-Ruiz, M. A modified colorimetric method for the determination of orthophosphate in the presence of high ATP concentrations. *Anal. Biochem.* **200**, 235-8 (1992).
5. Garrigues, A., Nugier, J., Orlowski, S. & Ezan, E. A high-throughput screening microplate test for the interaction of drugs with P-glycoprotein. *Anal. Biochem.* **305**, 106-114 (2002).
6. Kaur, H. et al. The ABC exporter MsbA probed by solid state NMR - challenges and opportunities. *Biol. Chem.* **396**, 1135-49 (2015).
7. Westfahl, K.M., Merten, J.A., Buchaklian, A.H. & Klug, C.S. Functionally Important ATP Binding and Hydrolysis Sites in Escherichia coli MsbA. *Biochemistry* **47**, 13878-13886 (2008).
8. Zou, P., Bortolus, M. & Mchaourab, H.S. Conformational cycle of the ABC transporter MsbA in liposomes: detailed analysis using double electron–electron resonance spectroscopy. *J. Mol. Biol.* **393**, 586-597 (2009).

9. Zou, P. & McHaourab, H.S. Alternating Access of the Putative Substrate-Binding Chamber in the ABC Transporter MsbA. *J. Mol. Biol.* **393**, 574-585 (2009).
10. Buchaklian, A.H., Funk, A.L. & Klug, C.S. Resting state conformation of the MsbA homodimer as studied by site-directed spin labeling. *Biochemistry* **43**, 8600-6 (2004).
11. Woebking, B. et al. Functional role of transmembrane helix 6 in drug binding and transport by the ABC transporter MsbA. *Biochemistry* **47**, 10904-10914 (2008).
